# Supplementary material for: Development and testing of a reduced carbohydrate intervention for the management of obesity and reduction of gestational diabetes (RECORD): protocol for a feasibility randomised controlled trial
Source: BMJ Open. 2022 Sep 1;12(9):e060951. doi: 10.1136/bmjopen-2022-060951 (PMC10441568; doi:10.1136/bmjopen-2022-060951)
Supplement: Author's manuscript [file bmjopen-2022-060951.draft_revisions.pdf]

BMJ Open

BMJ Open is committed to open peer review. As part of this commitment we make the peer review history of every article we publish publicly available.

When an article is published we post the peer reviewers' comments and the authors' responses online. We also post the versions of the paper that were used during peer review. These are the versions that the peer review comments apply to.

The versions of the paper that follow are the versions that were submitted during the peer review process. They are not the versions of record or the final published versions. They should not be cited or distributed as the published version of this manuscript.

BMJ Open is an open access journal and the full, final, typeset and author-corrected version of record of the manuscript is available on our site with no access controls, subscription charges or pay-per-view fees (http://bmjopen.bmj.com).

If you have any questions on BMJ Open's open peer review process please email info.bmjopen@bmj.com

BMJ Open

Reduced carbohydrate intervention for the management of obesity and reduction of gestational diabetes (RECORD): protocol for a feasibility randomised controlled trial

Journal:	*BMJ Open*
Manuscript ID	bmjopen-2022-060951
Article Type:	Protocol
Date Submitted by the Author:	08-Jan-2022
Complete List of Authors:	Michalopoulou, Moscho; University of Oxford, Nuffield Department of Primary Care Health Sciences; NIHR Oxford Biomedical Research Centre<br>Jebb, Susan; University of Oxford, Nuffield Department of Primary Care Health Sciences; NIHR Oxford Biomedical Research Centre<br>MacKillop, Lucy; University of Oxford Nuffield Department of Women's & Reproductive Health; Oxford University Hospitals NHS Foundation Trust<br>Dyson, Pamela; University of Oxford, Oxford Centre for Diabetes, Endocrinology and Metabolism (OCDEM), ; NIHR Oxford Biomedical Research Centre<br>Hirst, Jane E; University of Oxford, Nuffield Department of Women's and Reproductive Health; Oxford University Hospitals NHS Foundation Trust<br>Wire, Amy; Berkshire Healthcare NHS Foundation Trust<br>Astbury, Nerys; University of Oxford, Nuffiled Department of Primary Care Health Sciences; NIHR Oxford Biomedical Research Centre
Keywords:	Diabetes in pregnancy < DIABETES & ENDOCRINOLOGY, NUTRITION & DIETETICS, OBSTETRICS

SCHOLARONE™
Manuscripts

Reduced carbohydrate intervention for the management of obesity and reduction of gestational diabetes (RECORD): protocol for a feasibility randomised controlled trial

Moscho Michalopoulou,[1,5] Susan A Jebb,[1,5] Lucy Mackillop,[2,3] Pamela Dyson,[4,5] Jane E Hirst,[2,3] Amy Wire,[6] Nerys M Astbury[1,5]

[1] Nuffield Department of Primary Care Health Sciences, University of Oxford, United Kingdom

[2] Nuffield Department of Women's and Reproductive Health, University of Oxford, United Kingdom

[3] Oxford University Hospitals NHS Foundation Trust, United Kingdom

[4] Oxford Centre for Diabetes Endocrinology & Metabolism (OCDEM), United Kingdom

[5] National Institute for Health Research (NIHR) Oxford Biomedical Research Centre, United Kingdom

[6] Patient Representative

Corresponding Author: Moscho Michalopoulou, Nuffield Department of Primary Care Health Sciences, Radcliffe Primary Care Building, Woodstock Road, Oxford, OX2 6GG, email: moscho.michalopoulou@phc.ox.ac.uk

Word count: 4501

For peer review only

Abstract

Introduction:

Previous trials of dietary interventions to prevent gestational diabetes mellitus (GDM) have yielded only limited success. Low-carbohydrate diets have shown promise for the treatment of type 2 diabetes, but there is no evidence to support their use in pregnancy. The aim of this study is to explore the feasibility of a reduced-carbohydrate dietary intervention delivered from mid-pregnancy alongside routine antenatal care.

Methods and analysis:

This is a feasibility randomised controlled trial (RCT) with embedded qualitative study. Sixty women who are pregnant <20 weeks' gestation, with body mass index ≥30 kg/m^2 at their antenatal booking appointment, will be randomised 2:1 intervention or control (usual care) and followed-up until delivery. The intervention is a moderately reduced-carbohydrate diet (~130-150g total carbohydrate/day), designed to be delivered alongside routine antenatal appointments. Primary outcomes are measures of adoption of the diet and retention of participants. Secondary outcomes include process and qualitative measures. Exploratory outcomes include incidence of GDM, change in markers of glycaemic control, dietary intake, gestational weight gain, quality of life, and maternal and neonatal outcomes.

Ethics and dissemination:

This trial was reviewed and approved by the South-Central Oxford B Research Ethics Committee NHS National Research Ethics Committee and the Health Research Authority (Reference: 20/SC/0442). The study results will inform whether to progress to a full-scale RCT to test the clinical effectiveness of the RECORD programme to prevent GDM in women at high risk. The findings will be published in peer reviewed journals and presented at conferences.

Trial registration: ISRCTN1623588

For peer review only

Strengths and limitations of this study

- This study will test the adoption of a novel reduced-carbohydrate intervention to reduce the risk of gestational diabetes and assess its suitability to progress to a full trial.

- The intervention has been developed by a multidisciplinary team of academic researchers and clinical professionals working in maternity, together with patients, to create a support programme likely to be acceptable to women and suitable for use in routine antenatal care.

- This study will provide new evidence of the effect of a reduced-carbohydrate intervention on capillary glucose and ketone levels, and the natural progression of these markers in the control group, from mid-pregnancy until delivery.

- This study is not powered to detect clinical effectiveness of the dietary intervention, and subsequent full-scale trials will be required to assess whether this intervention can effectively attenuate gestational weight gain and improve glycaemic control early in pregnancy, with the prospect of preventing gestational diabetes.

For peer review only

For peer review only - http://bmjopen.bmj.com/site/about/guidelines.xhtml

Introduction

Gestational diabetes mellitus (GDM), is defined as hyperglycaemia that develops or is first diagnosed during pregnancy.[1] GDM significantly increases the risk for adverse health outcomes for both the mother and the offspring,[2,3-9] In the UK, approximately 16% of women who are pregnant develop GDM, but the prevalence reaches up to 25-30% in women living with obesity.[10] Excessive gestational weight gain (GWG) has also been proposed as a risk factor for GDM,[11] and several clinical trials in women with overweight or obesity during pregnancy, have tested whether interventions aimed at limiting GWG could help prevent GDM.[12 13] Although some trials have been successful in attenuating GWG, the majority have failed to demonstrate a reduced risk of developing GDM.

The small number of trials that have shown effectiveness in GDM prevention so far included intensive face-to-face dietetic support and/or provision of key foods to study participants, with no indication of how these strategies could be delivered in routine practice. For example, the RADIEL study, reduced the incidence of GDM by 39% in women at high risk, but required extra face-to-face visits and a group session to deliver standard healthy eating and physical activity advice.[14] The ESTEEM and St Carlos trials, implemented a Mediterranean-style diet supplemented with extra virgin olive oil and nuts, provided as part of the trial, from early or mid-pregnancy, and showed a similar reduction in GDM risk.[15 16] Nevertheless, wide scale implementation of a Mediterranean-style diet in the UK could be challenging due to limited understanding of the dietary pattern, differences in dietary habits and preferences, and the cost of extra virgin olive oil.[17-19]

The mechanism behind the success of some dietary interventions over others in preventing GDM remains unclear. Implementation at earlier stages of pregnancy seems to be key, but the optimum dietary composition is unknown. In the RADIEL study, there was only a small improvement reported in adherence to the diet recommendations in the intervention group compared to the control group.[14] In the Mediterranean dietary interventions, it was speculated that increased

For peer review only - http://bmjopen.bmj.com/site/about/guidelines.xhtml

unsaturated fat and polyphenol consumption might have been beneficial with regards to insulin sensitivity and inflammation.[15 16] However, a reduction in dietary carbohydrate as a natural result of higher fat and protein consumption, in conditions when energy intake remains similar, may have also played a role. Indeed, successful interventions to date included some advice for reducing sugar consumption and/or moderating starchy foods.

Low-carbohydrate are effective for the management of type 2 diabetes, especially over the short-term (3-6 months).[20] Reduced carbohydrate intake from earlier stages of pregnancy may help reduce hyperglycaemia, independent of effects on weight gain, and may also help attenuate GWG by reducing energy intake. Although low-carbohydrate diets (<130g/day),[21] are not recommended for women who are pregnant, due to safety concerns associated with ketosis, research in non-pregnant populations suggests that significant ketosis only occurs when carbohydrate intake is severely restricted to <50g/day, leaving considerable scope to adopt a reduced-carbohydrate diet.[21] One previous randomised controlled trial specifically aimed at reducing total carbohydrate consumption as part of an intensive healthy eating intervention.[22] Despite a substantial significant effect on limiting GWG, the intervention did not reduce GDM incidence. This study was underpowered to detect difference in GDM risk, and it resulted in only a small reduction in carbohydrate intake compared to usual care (reduction of 4.8% of energy intake from carbohydrates, equivalent to about 30g of carbohydrate per day). Thus, the effect of a reduced-carbohydrate diet as a preventative strategy for GDM remains to be investigated.

We have developed a dietary approach based on moderate carbohydrate reduction suitable for use in pregnancy, combined with behavioural support. The reduced-carbohydrate intervention for the management of obesity and reduction of gestational diabetes (RECORD), also recommends consumption of fresh foods, plenty of vegetables, and moderate protein intake. It was designed with midwives and dietitians experienced in working with women who are pregnant, together with patients, and is designed to be delivered alongside routine antenatal appointments. The aim of this

For peer review only - http://bmjopen.bmj.com/site/about/guidelines.xhtml

trial is to assess the feasibility of the intervention and whether it leads to a significant reduction in carbohydrate intake. Other objectives include: a) assess the study procedures to inform the design of a full randomised controlled trial; b) to qualitatively analyse participant experiences of the intervention; c) to explore the potential effect of the reduced-carbohydrate behavioural intervention on markers of glycaemic control, incidence of GDM, GWG, dietary intake, quality of life, and maternal and neonatal outcomes.

Study design

A two-arm parallel group individually randomised controlled trial (RCT), in women who are at <20 weeks' gestation with a BMI ≥30kg/m². Participation will last approximately 6 months per participant; from early in the second trimester (no more than 19 weeks plus 6 days), until delivery. Participants will attend two study visits, one at baseline (<20 weeks' gestation) and one for follow-up, at 24-28 weeks' gestation.

Recruitment

Participants will be recruited from one maternity care centre in Oxfordshire, UK. The study is expected to run from May 2021 to December 2022. Women attending the dating or nuchal ultrasound scan appointment (typically between 8 and 14 weeks' gestation) will be approached to take part in the study. Potentially eligible participants will be provided with the participant information sheet, and invited to give their contact details. A member of the research team will contact the women who were interested in taking part to discuss the study and assess self-reported eligibility to participate according to the full inclusion and exclusion criteria as detailed below. Eligible individuals who wish to participate will be booked in for a face-to-face baseline appointment. For those who decline to participate, permission will be sought to record the barriers to participation.

For peer review only - http://bmjopen.bmj.com/site/about/guidelines.xhtml

For peer review only

Eligibility criteria

Inclusion criteria

- Pregnant women <20 weeks' gestation.
- Aged ≥18 years.
- BMI≥30kg/m^2 measured at booking appointment.
- Planned antenatal care and delivery at the study setting.

Exclusion criteria

The participant may be excluded if any of the following apply:

- Severe congenital fetal anomaly (confirmed by ultrasound).
- Planned termination of pregnancy or ectopic pregnancy.
- Pre-pregnancy diagnosis of diabetes or impaired glucose tolerance or taking metformin.
- Renal disease.
- Severe liver disease.
- Organ transplant.
- Cardiac failure (Grade II New York Heart Association, and more severe).[23]
- Severe neurological disorder (including epilepsy).
- Severe psychiatric disease requiring in-patient admission.
- GDM diagnosis at the baseline study visit according to the National Institute for Health and Care Excellence (NICE) diagnostic criteria: a fasting plasma glucose level of 5.6mmol/L or above, or a 2-hour plasma glucose level of 7.8mmol/L and above.[24]
- History or presence of eating disorder.
- Hyperemesis gravidarum.
- Unable to understand spoken and written English.

For peer review only

For peer review only - http://bmjopen.bmj.com/site/about/guidelines.xhtml

- Previous bariatric surgery.
- Women participating in other intervention research studies.
- Any other significant disease or disorder which may either put the participants at risk because of participation in the trial, or may influence the result of the study, or the participant's ability to participate in the study.

Study procedures

The baseline visit will take place before 20 weeks' gestation, at the hospital, with a member of the research team, and lasts for approximately 2 hours. Written informed consent will be sought and eligibility for inclusion in formally assessed. All participants will complete the following baseline assessments: blood pressure, weight and height measurements, a two-hour 75 gram Oral Glucose Tolerance Test (OGTT), a quality of life questionnaire, and measurement of their carbohydrate intake as part of a broader assessment of their dietary intake via an online questionnaire. The participants will be randomised to one of the two trial arms. Participants in the intervention group will proceed to receive the intervention session. Due to the nature of the OGTT test and exclusion of women with GDM confirmed on this baseline test, full eligibility can only be confirmed after the visit, therefore after randomisation. All participants will be invited to attend a further 2-hour research visit at 24-28 weeks' gestation where the same research measurements are collected as a follow-up, and a second OGTT is completed. Maternal and neonatal outcomes will be extracted from medical records after delivery. The study flow is in **Figure 1**.

Sample size

This feasibility study is not powered to detect a statistically significant difference in clinical effectiveness between the trial arms. Instead, we will test for reductions in carbohydrate intake as preliminary evidence of the adoption of the reduced carbohydrate diet to an extent that is expected to alter health outcomes. The average intake in women who are not pregnant is about 230g/day.[25]

For peer review only

For peer review only - http://bmjopen.bmj.com/site/about/guidelines.xhtml

We have set the sample size to be able to detect a reduction to 130-150g of total carbohydrate/day at follow-up with 95% Confidence Interval (CI) excluding 170g/day. Progression criteria to a full trial also include adequate retention of participants as a measure of feasibility. We consider that a minimum of 80% of participants need to complete an OGTT at 24-28 weeks' gestation, similar to previous trials, with 95% CI excluding 70%.[15 26] Both progression criteria thresholds are satisfied with a sample size of 60 participants allocated in 2:1 ratio, intervention: control. Unequal randomisation allows us to have sufficient participants in the intervention arm for the qualitative study.

Randomisation

Eligible participants will be randomised to one of the two treatment groups using permuted block randomisation with random block sizes of 3 and 6. An independent researcher will generate the allocation sequence and assignment will be revealed once eligibility has been confirmed using an online software (Study Randomizer) to ensure allocation concealment.[27] Due to the nature of this study, it will not be possible to blind the participants, clinicians or some of the study team to the treatment allocation beyond this point.

Intervention and control

Intervention: the RECORD programme

The intervention comprises a moderately reduced-carbohydrate behavioural intervention, combining dietary advice, with structured written dietary information. It aims to improve participants' adherence to the programme through a simple behavioural support programme, which includes goal-setting, planning, feedback and problem-solving. The intervention will be delivered during a 30-minute session with a healthcare professional (HCP) during the baseline visit, where the intervention materials and advice will be provided, and will be supplemented with up to 6 brief telephone support sessions of up to 15 minutes depending on patient need. Participants in this group will be advised to follow the intervention until delivery.

For peer review only - http://bmjopen.bmj.com/site/about/guidelines.xhtml

This intervention was developed based on a review of the literature, study protocols, qualitative data of similar interventions, and interviews with a diabetes-specialist dietitian, two obstetricians, a research midwife, and a patient representative with previous GDM. A group of women affected by GDM, also helped to shape the ideas for this research, and to determine the questions we are trying to answer, and the type of dietary information and support they felt would be helpful.

The dietary component of this intervention consists of a moderately reduced-carbohydrate diet (130-150g of total carbohydrate per day), with no additional energy restriction. This is in line with the dietary advice provided in previous GDM trials, as well as for women diagnosed with GDM at the hospital. The difference here, is that the reduced-carbohydrate advice is given earlier in pregnancy, to assess whether it has benefits for women at increased risk of GDM. The core principles include:

a) **REFRAIN** from having sugary foods and drinks (e.g. biscuits, confectionary, juice, sugary fizzy drinks), with the exception of dairy and limited fruit intake;

b) **REDUCE** the amount of starchy carbohydrates to a moderate level, by having smaller portions of foods such as rice, pasta, bread, grains, potato, yams, cassava, plantain, at main meals;

c) **REPLACE** refined starchy carbohydrates with wholegrain, unrefined versions, and sugary foods and drinks, with lower-carbohydrate, and lower, or no-sugar alternatives.

Standard healthy eating advice during pregnancy is also included, e.g. high intake of vegetables, moderate intake of lean protein, and guidance on foods that should be avoided during pregnancy. Information is discussed regarding the importance of controlling GWG, and women in this group are advised to weigh themselves once a week.

The development of this intervention was also informed by behavioural frameworks aiming to promote successful behaviour change. These are: the Capability, Opportunity, and Motivation-

For peer review only

For peer review only - http://bmjopen.bmj.com/site/about/guidelines.xhtml

Behavioural model (COM-B), the Theoretical Domains Framework (TDF), and the Behaviour Change Wheel (BCW).[28 29] By utilising these frameworks, we targeted the components of psychological capability (e.g. knowledge/understanding, skills, decision making, self-regulation), physical and social opportunity (e.g. social influences and support, and environmental changes), and reflective motivation (e.g. knowledge/understanding, confidence regarding capabilities, beliefs regarding risks, intentions, plans). To address these components, the intervention includes:

- A consultation including the essential principles of the diet as described above, as well as the rationale behind the intervention and how it may work in controlling blood glucose levels, and attenuating GWG.
- Written resources to enhance participants' confidence and skills in following the programme, and to facilitate decision making, including advice on food selection, meal and snack suggestions, and suggested recipes.
- Setting personalised realistic goals, by letting participant choose what changes they are comfortable making and the steps to achieve them. Participants can choose to use a "Personal action plan" booklet to set their goals and reflect on their progress, as well as goal progression checklists, and self-weighing charts, however, these are also addressed during the support sessions.
- Structured healthcare professional follow-up to provide support, contingency planning, problem-solving, planning of social support, advice on lapses, and help with environmental or emotional triggers, and to provide feedback on progress. Additionally, participants are able to self-monitor their health via home finger-prick testing of their blood glucose and ketones, and by weighing themselves, which provides additional monitoring.
- Provision of feedback on progress and changes in clinical measures as a result of following the intervention, e.g. GWG, capillary blood glucose and ketones, glucose curve from OGTTs, blood pressure.

For peer review only - http://bmjopen.bmj.com/site/about/guidelines.xhtml

Control

All women will receive routine antenatal care, which usually includes one-off face-to-face standard healthy eating advice during their booking appointment with a midwife. This will have been given prior to randomisation in the study. Participants allocated to the control group will receive no additional advice beyond this.

Patient and public involvement

We convened two focus groups with eight women recruited from the local antenatal clinic, national GDM support website, and a social media forum. The first group helped to prioritise and refine the research question. In a second phase, women were provided with an outline of the study proposal, and were asked to provide further comments. From both phases, it was clear that women felt upset, shocked and anxious following a diagnosis of GDM, and highlighted a need for preventative interventions that could be implemented earlier in pregnancy. Participants also highlighted the importance of including neonatal outcomes in addition to the maternal outcomes. Women were interested in taking part in clinical trials aiming to prevent, treat or manage GDM, however, felt that that attending extra appointments for study visits and in particular having to use the parking at the hospital, would be a major barrier to participation. As a final stage, a patient representative provided feedback on the intervention materials to help make them clearer and more concise and continues to support the study team.

Outcomes

Primary

The primary objective of this study is to test whether this reduced carbohydrate programme is feasible and leads to a reduction in carbohydrate intake in women who are pregnant and at risk of developing GDM. This feasibility measures will further determine whether to progress to a full RCT.

For peer review only - http://bmjopen.bmj.com/site/about/guidelines.xhtml

Progression criteria will be based on a traffic light system to decide whether to proceed to a full RCT (green), proceed with amendments for remediable issues (amber), or to not to proceed (red). (**Figure 2**)[30] The progression criteria will be:

1. **Adoption of the intervention:** that the intervention group reduces their total carbohydrate intake by the time of follow-up to at least 170g/day. (Evaluated as part of a broader assessment of dietary intake assessment through an online 24-hour dietary recall questionnaire – detailed below).
2. **Retention rate:** that at least 70% of all participants complete an OGTT at the follow-up time point. (Evaluated by documentation at 24-28 weeks' gestation; assessed as the proportion of participants for whom OGTT measurements are available).

Secondary

Secondary outcomes will examine procedures, resources and management issues, and assess the acceptability of the intervention, to aid the design of a future RCT.

Process:

- Number of eligible women who are approached and enrolled into the study per month.
- Percentage of intervention participants who complete the initial and support sessions.
- Percentage of participants in each group who attend the follow-up visit.
- Percentage of participants for whom weight at around 36 weeks' gestation is available (usually measured as part of a routine hospital appointment).
- Proportion of participants who record their blood glucose and blood ketones every week.
- Proportion of participants in the intervention group who self-monitor their weight every week.
- Proportion of participants in the control group who, after randomisation, follow a reduced-carbohydrate diet (contamination of the control group).

For peer review only - http://bmjopen.bmj.com/site/about/guidelines.xhtml

- Proportion of essential elements included in intervention delivery sessions (main and support sessions).

Qualitative:

- Participant acceptability of the intervention and the materials, overall experience of the intervention, barriers and facilitators of adherence (qualitative interviews).

Exploratory outcomes:

- Distribution of fasting capillary blood glucose and ketone levels across gestational age.
- Change in fasting plasma glucose, 1-hour and 2-hour glucose (OGTT).
- Change in HbA1c, fasting plasma insulin and insulin resistance (HOMA-IR).(ref)
- Proportion of participants who are diagnosed with GDM.
- GWG: weight change from before 20 weeks' gestation until around 36 weeks' gestation.
- Proportion of participants meeting the Institute of Medicine recommendations for GWG.[31]
- Changes in dietary intake.
- Changes in systolic and diastolic blood pressure.
- Changes in pregnancy-related outcomes e.g. gestational hypertension and preeclampsia, mode of delivery, birthweight, neonatal adverse events.
- Change in quality of life.

Measurements

A schedule of measurements can be found in **Table 1**.

Sociodemographic characteristics

Participants will be asked to self-report highest education level, employment status and ethnicity. Age will be accessed from the medical records.

For peer review only - http://bmjopen.bmj.com/site/about/guidelines.xhtml

Physical measurements

Height will be measured using stadiometers, to the nearest 0.1 cm. Weight will be measured to the nearest 0.1kg using a digital scale (Active Era, BS-03S) at the study visits. For self-weighing at home, participants in the intervention group will be instructed to use their own digital scales or will be given a set by the research team if they do not own a set. They can either text the measurements to the research team or log them manually onto a mobile application (detailed below). At 36-weeks' gestation, women should be weighed during a routine antenatal appointment at their primary care practice, and digital scales are used for this. Blood pressure will be measured in duplicate using a digital blood pressure machine (Omron, M3) after 5 min seated rest, with at least 1 min between each measurements.

OGTT and remote glucose and ketone monitoring

Women are asked to attend the study visits after an overnight fast, and to refrain from smoking, vaping, using nicotine replacement therapy or chewed sugary gum for at least 8 hours before. A fasting venous blood sample will be collected for the determination of glucose, HbA1c, insulin concentrations. Participants are provided with a 75g glucose load (Polycal, Nutricia). Further venous blood samples will be collected at 1 hour and 2 hours post-drink, for the determination of glucose concentrations.

All participants are provided with a Bluetooth-enabled capillary blood glucose and ketone monitoring kit (4SURE Smart Duo, Nipro Diagnostics Ltd). The researcher provides a short demonstration and information flyer on how to collect and record the measurements, and advises participants to take a fasting capillary glucose and ketone measurement twice a week. The monitor is connected remotely to a mobile application (Diabetes: M, Sirma Medical Systems), allowing the research team to monitor the readings remotely and escalate concerns to the clinical team.

For peer review only

For peer review only - http://bmjopen.bmj.com/site/about/guidelines.xhtml

Carbohydrate consumption

Participants will complete an online multiple-pass 24-hour recall, validated against interviewer-led multiple-pass recalls in adolescents and adults (Intake 24, Newcastle University).[32 33] This will be used to primarily assess carbohydrate consumption, but also broader dietary intake.

Quality of life

Participants will be asked to fill in a questionnaire about their quality of life, via the European Quality of Life Five Dimension questionnaire and subscale.[34]

Retention and withdrawal

This study has been designed to run alongside routine antenatal appointments with remote support elements, to minimise the burden associated with attending extra visits. The brief telephone support sessions with a dietitian will act as prompts for the participants to engage with the intervention and self-monitoring. Participants are asked to attend one visit in addition to routine antenatal care. All of the women who take part would be offered an OGTT at 24-28 weeks of pregnancy for the diagnosis of GDM. The 24-28-week follow-up study visit and OGTT, replaces the routine care OGTT, and the results are shared with the clinical care team. To compensate for the time and inconvenience in taking part, we offer participants financial incentives for their participation.

Each participant will have the right to withdraw from the study at any time, or may be withdrawn at the discretion of the research or clinical care team. Participants will have the right to withdraw their collected data if they wish. Withdrawn eligible and enrolled participants will not be replaced in this feasibility study. We will only replace participants whose baseline OGTT is abnormal, as they will have consented and been randomised before OGTT results become available, but they will have essentially failed the full screening.

For peer review only - http://bmjopen.bmj.com/site/about/guidelines.xhtml

GDM diagnosis

Participants who develop GDM or who are diagnosed with GDM at the 24-28 weeks' follow-up visit, will be referred to the clinical care team and offered the current standard medical treatment. In line with the NICE guidelines, this includes monitoring of blood glucose (two of the fasting measurements will continue to be used in the study), and diet and physical activity advice for the management of GDM.[24] More specifically, women with GDM are advised to manage carbohydrate intake, to limit their weight gain until delivery, and are encouraged to do 150 minutes of moderate intensity activity per week. Women in the intervention group, can choose to continue following the RECORD programme until delivery (at the discretion of the clinical care team). Women in the control group will receive advice to reduce their carbohydrate intake at this stage, and this will be noted. All participants with GDM, will be encouraged to continue monitoring their ketone levels twice a week for the remaining of pregnancy.

Statistical analysis

Descriptive statistics will be used to report the baseline characteristics of each trial arm. Continuous variables will be reported using mean/median and standard deviation/range, while categorical variables will be reported using frequencies and percentages.

Since this is a feasibility study, the primary outcomes are the main progression criteria which will inform the design of a future RCT, powered to detect a significant difference in the efficacy of the intervention in preventing GDM. Descriptive statistics with 95% CIs will be used to analyse and report the progression criteria. For the analysis of the secondary, process-related and exploratory measures, all data collected up to each time point will be used. Descriptive comparative statistics will be used to analyse and report these measures along with 95% CIs (e.g. difference in means or proportions between baseline and follow-up for each group or between the two groups). Data from

For peer review only

For peer review only - http://bmjopen.bmj.com/site/about/guidelines.xhtml

audio-recorded and transcribed qualitative interviews on acceptability and experience of the intervention by participants, will be analysed using thematic analysis and reported descriptively.

Qualitative study

A qualitative sub-study with participants randomised to the RECORD group will explore participants' acceptability of the intervention and their overall experience of the study, explore barriers and facilitators to adherence, and identify suggestions that could refine the intervention should it progress to a full RCT.

During the follow-up study visit a member of the research team will provide participants allocated to the intervention group, with a separate participant information sheet about the qualitative interviews. If participants are happy to have this in-depth discussion about their experiences of the RECORD intervention, a member of the research team will arrange a convenient day and time for a telephone interview. The participants will consent verbally on the phone and the researcher will fill in written consent form. The interviews will last approximately 30 minutes and all interviews will be audio-recorded and transcribed verbatim. Data will be analysed using thematic analysis. We will conduct interviews with up to 30 participants, or until data saturation is reached.

Trial management and monitoring

The day-to-day management of the study will be coordinated by MM. As this is a small feasibility study, with no adverse event monitoring or stopping rules, there are no monitoring committees in place. A trial management group consisting of the authors of this paper, a research midwife, and a patient representative, will have oversight of the trial and meet bi-monthly to discuss progress and decide on actions to be taken.

For peer review only - http://bmjopen.bmj.com/site/about/guidelines.xhtml

Adverse events

We will record and report all serious adverse events (SAEs) according to Good Clinical Practice (GCP), and Health Research Authority's (HRA) processes. Recording of SAEs will start immediately following enrolment into the study and will be continued until the end of the study.

Data management

Data will be kept in accordance with GCP, the Data Protection Act 2018, and the General Data Protection Regulation. Two separate databases will be created, one containing all participant identifiable information, and one where data collected during the research procedures will be entered in an anonymised manner, using a unique participant ID (electronic CRFs). Anonymised data from the online diet questionnaire will be downloaded from the Intake24 website and added to the second database. Quality of life anonymised data will be collected on hard copy questionnaires and then entered into the second database. The two databases, as well as anonymised recordings and transcriptions from the main and support intervention sessions, will be password-protected and stored on the secure drive of Nuffield Department of Primary Care Health Sciences, and will only be accessible to authorised members of the research team. After publication of the results has been sent out to participants, their contact details will be deleted/destroyed. We will retain the anonymised research data for future secondary analyses.

Direct access to study data will be granted to authorised representatives from the Sponsor and host institution for monitoring and/or audit of the study to ensure compliance with regulations. Otherwise, confidentiality will be maintained and no-one outside the research team will have access to the database.

For peer review only

For peer review only - http://bmjopen.bmj.com/site/about/guidelines.xhtml

Ethics and dissemination

This protocol (Version 1.3, 12 July 2021) was reviewed and approved by the South-Central Oxford B Research Ethics Committee NHS National Research Ethics Committee and the Health Research Authority (Reference: 20/SC/0442), and prospectively registered on ISRCTN (ISRCTN16235884). Any substantial changes to the protocol will be notified and reviewed by NHS Research Ethics Committee, the Health Research Authority and the Sponsor (University of Oxford), and trial registry will be updated accordingly. The findings of this feasibility study will be submitted for publication in a peer-reviewed journal, and presented at conferences, to disseminate the results to academic and health professional audiences, and made available to participants and to the wider public on our website at the time of publication.

For peer review only

Sponsor: This trial is sponsored by the University of Oxford, Clinical Trials and Research Governance, Joint Research Office, Block 60, Churchill Hospital, Old Road, Headington, Oxford, OX3 7LE, UK.

Funding: This trial is funded by the NIHR Oxford Biomedical Research Centre. MM's time on this project is funded by Oxford-Medical Research Council Doctoral Training Partnership. JEH is supported by a UK Research and Innovation Future Leaders Fellowship.

Disclaimer: The views expressed are those of the authors and not necessarily those of the NHS, the NIHR, the University of Oxford or the Department of Health. The sponsor or funding bodies had no role in the study design, collection, management, analysis, and interpretation of data; writing of the report; and the decision to submit the report for publication.

For peer review only

Contributions: MM, NA and SAJ developed the concept for the study and wrote the first draft of the protocol with input from LM and JEH on clinical aspects. MM, NA and SAJ prepared the study documents and coordinated the HRA and ethics application. The sponsor has reviewed all participant-facing documents as part of the ethics application. LM, JEH and PD approved the final protocol. MM, NA, SAJ and PD were involved in the detailed design of the intervention. MM and PD developed the intervention materials with input from NA, SAJ, LM and JEH. MM drafted the manuscript for publication, with input from NA and SAJ. LM, JEH and PD reviewed, provided input and approved the final manuscript.

Competing interests:

LM is a part-time employee and shareholder of Sensyne Health PLc.

Acknowledgements:

We thank Sr Yvonne Kenworthy, Research Midwife, for providing feedback on the intervention materials.

For peer review only - http://bmjopen.bmj.com/site/about/guidelines.xhtml

Table 1. Schedule of study measurements

	STUDY PERIOD								
	**Enrolment**	**Allocation**	**Study visits/sessions**						**Delivery**
**TIMEPOINT**	Baseline = Week 0 (<20 weeks' gestation)	Week 0	Week 2	Week 4	~Week 8	Follow-up visit = ~Week 12 (24-28 weeks' gestation)	~Week 16	~Week 20	Week 24
**ENROLMENT:**									
**Eligibility screening**	X								
**Informed consent**	X								
**Randomisation**	X								
**TRIAL ARMS:**									
**RECORD programme**	X	X				X			X
**+ telephone support**			X	X	X	X	X	X	
**Usual care**	X	X				X			X
**ASSESSMENTS:**									
**Demographics**	X								
**Weight**	X					X			
**Height**	X								
**Blood pressure**	X					X			
**OGTT**	X					X			
**EQ-5D**	X					X			
**Self-completed 24-hour dietary recall**	X					X			
**Fasting plasma insulin**	X					X			
**HbA1c**	X					X			
**Self-monitoring of fasting capillary glucose**		Twice a week for the study duration							
**Self-monitoring of fasting capillary ketones**		Twice a week for the study duration							
**Self-weighing (intervention group)**		Once a week for the study duration							
**SAEs**									
**Maternal and neonatal outcomes**									X

EQ-5D: EuroQol – 5 Dimensions questionnaire; OGTT: Oral Glucose Tolerance Test; SAEs: Serious Adverse Events

For peer review only

For peer review only - http://bmjopen.bmj.com/site/about/guidelines.xhtml

References

1. American Diabetes Association. 2. Classification and Diagnosis of Diabetes: Standards of Medical Care in Diabetes-2020. *Diabetes Care* 2020;43(Suppl 1):S14-S31. doi: 10.2337/dc20-S002 [published Online First: 2019/12/22]
2. The HAPO Study Cooperative Research Group, Metzger BE, Lowe LP, et al. Hyperglycemia and adverse pregnancy outcomes. *N Engl J Med* 2008;358(19):1991-2002. doi: 10.1056/NEJMoa0707943 [published Online First: 2008/05/09]
3. Vounzoulaki E, Khunti K, Abner SC, et al. Progression to type 2 diabetes in women with a known history of gestational diabetes: systematic review and meta-analysis. *BMJ* 2020;369:m1361. doi: 10.1136/bmj.m1361 [published Online First: 2020/05/15]
4. Daly B, Toulis KA, Thomas N, et al. Increased risk of ischemic heart disease, hypertension, and type 2 diabetes in women with previous gestational diabetes mellitus, a target group in general practice for preventive interventions: A population-based cohort study. *PLoS Med* 2018;15(1):e1002488. doi: 10.1371/journal.pmed.1002488 [published Online First: 2018/01/18]
5. Lowe WL, Jr., Lowe LP, Kuang A, et al. Maternal glucose levels during pregnancy and childhood adiposity in the Hyperglycemia and Adverse Pregnancy Outcome Follow-up Study. *Diabetologia* 2019;62(4):598-610. doi: 10.1007/s00125-018-4809-6 [published Online First: 2019/01/17]
6. Kramer CK, Campbell S, Retnakaran R. Gestational diabetes and the risk of cardiovascular disease in women: a systematic review and meta-analysis. *Diabetologia* 2019;62(6):905-14. doi: 10.1007/s00125-019-4840-2 [published Online First: 2019/03/08]
7. Boney CM, Verma A, Tucker R, et al. Metabolic syndrome in childhood: association with birth weight, maternal obesity, and gestational diabetes mellitus. *Pediatrics* 2005;115(3):e290-6. doi: 10.1542/peds.2004-1808 [published Online First: 2005/03/03]
8. Yu Y, Arah OA, Liew Z, et al. Maternal diabetes during pregnancy and early onset of cardiovascular disease in offspring: population based cohort study with 40 years of follow-up. *BMJ* 2019;367:l6398. doi: 10.1136/bmj.l6398 [published Online First: 2019/12/06]
9. Lowe WL, Jr., Scholtens DM, Kuang A, et al. Hyperglycemia and Adverse Pregnancy Outcome Follow-up Study (HAPO FUS): Maternal Gestational Diabetes Mellitus and Childhood Glucose Metabolism. *Diabetes Care* 2019;42(3):372-80. doi: 10.2337/dc18-1646 [published Online First: 2019/01/19]
10. Farrar D, Simmonds M, Griffin S, et al. The identification and treatment of women with hyperglycaemia in pregnancy: an analysis of individual participant data, systematic reviews, meta-analyses and an economic evaluation. *Health Technol Assess* 2016;20(86):1-348. doi: 10.3310/hta20860 [published Online First: 2016/12/06]
11. Brunner S, Stecher L, Ziebarth S, et al. Excessive gestational weight gain prior to glucose screening and the risk of gestational diabetes: a meta-analysis. *Diabetologia* 2015;58(10):2229-37. doi: 10.1007/s00125-015-3686-5 [published Online First: 2015/07/05]
12. Griffith RJ, Alsweiler J, Moore AE, et al. Interventions to prevent women from developing gestational diabetes mellitus: an overview of Cochrane Reviews. *Cochrane Database Syst Rev* 2020;6:CD012394. doi: 10.1002/14651858.CD012394.pub3 [published Online First: 2020/06/12]
13. International Weight Management in Pregnancy Collaborative Group. Effect of diet and physical activity based interventions in pregnancy on gestational weight gain and pregnancy outcomes: meta-analysis of individual participant data from randomised trials. *BMJ* 2017;358:j3119. doi: 10.1136/bmj.j3119 [published Online First: 2017/07/21]
14. Koivusalo SB, Rönö K, Klemetti MM, et al. Gestational Diabetes Mellitus Can Be Prevented by Lifestyle Intervention: The Finnish Gestational Diabetes Prevention Study (RADIEL): A

For peer review only

Randomized Controlled Trial. *Diabetes Care* 2016;39(1):24-30. doi: 10.2337/dc15-0511 [published Online First: 2015/08/01]
15. B HAW, Dodds J, Placzek A, et al. Mediterranean-style diet in pregnant women with metabolic risk factors (ESTEEM): A pragmatic multicentre randomised trial. *PLoS Med* 2019;16(7):e1002857. doi: 10.1371/journal.pmed.1002857 [published Online First: 2019/07/25]
16. Assaf-Balut C, Garcia de la Torre N, Duran A, et al. A Mediterranean diet with additional extra virgin olive oil and pistachios reduces the incidence of gestational diabetes mellitus (GDM): A randomized controlled trial: The St. Carlos GDM prevention study. *PLoS One* 2017;12(10):e0185873. doi: 10.1371/journal.pone.0185873 [published Online First: 2017/10/20]
17. Bush H WR, Bradby H, Anderson A, Lean M. . Family hospitality and ethnic tradition among South Asian, Italian and general population women in the West of Scotland. *Social Health Ill* 1998;20(3):351-80.
18. Drewnowski A, Darmon N. Food choices and diet costs: an economic analysis. *J Nutr* 2005;135(4):900-4. doi: 10.1093/jn/135.4.900 [published Online First: 2005/03/30]
19. Moore SE, McEvoy CT, Prior L, et al. Barriers to adopting a Mediterranean diet in Northern European adults at high risk of developing cardiovascular disease. *J Hum Nutr Diet* 2018;31(4):451-62. doi: 10.1111/jhn.12523 [published Online First: 2017/11/22]
20. Singh M, Hung ES, Cullum A, et al. Lower carbohydrate diets for adults with type 2 diabetes. *Br J Nutr* 2021:1-6. doi: 10.1017/s0007114521002373 [published Online First: 2021/11/02]
21. Feinman RD, Pogozelski WK, Astrup A, et al. Dietary carbohydrate restriction as the first approach in diabetes management: critical review and evidence base. *Nutrition* 2015;31(1):1-13. doi: 10.1016/j.nut.2014.06.011 [published Online First: 2014/10/08]
22. Simmons D, Devlieger R, van Assche A, et al. Effect of Physical Activity and/or Healthy Eating on GDM Risk: The DALI Lifestyle Study. *J Clin Endocrinol Metab* 2017;102(3):903-13. doi: 10.1210/jc.2016-3455 [published Online First: 2016/12/10]
23. New York Heart Association. Inc. Diseases of the Heart and Blood Vessels. Nomenclature and Criteria for diagnosis. 6th ed. Boston1964:114.
24. NICE. Gestational diabetes: risk assessment, testing, diagnosis and management. Available from: https://pathways.nice.org.uk/pathways/diabetes-in-pregnancy/gestational-diabetes-risk-assessment-testing-diagnosis-and-management#content=view-node%3Anodes-risk-assessment. Accessed August 2021.
25. Roberts CS, Maplethorpe, N, Cox N, et al. National Diet and Nutrition Survey: Results from Years 7 and 8 (combined) of the Rolling Programme (2014/2015 to 2015/2016). London: Public Health England; 2018.
26. Poston L, Bell R, Croker H, et al. Effect of a behavioural intervention in obese pregnant women (the UPBEAT study): a multicentre, randomised controlled trial. *Lancet Diabetes Endocrinol* 2015;3(10):767-77. doi: 10.1016/S2213-8587(15)00227-2 [published Online First: 2015/07/15]
27. Study Randomizer. Available from: https://www.studyrandomizer.com/. Accessed May 2021.
28. Michie S, van Stralen MM, West R. The behaviour change wheel: a new method for characterising and designing behaviour change interventions. *Implement Sci* 2011;6:42. doi: 10.1186/1748-5908-6-42 [published Online First: 2011/04/26]
29. Michie S, Johnston M, Abraham C, et al. Making psychological theory useful for implementing evidence based practice: a consensus approach. *Qual Saf Health Care* 2005;14(1):26-33. doi: 10.1136/qshc.2004.011155 [published Online First: 2005/02/05]
30. Avery KN, Williamson PR, Gamble C, et al. Informing efficient randomised controlled trials: exploration of challenges in developing progression criteria for internal pilot studies. *BMJ Open* 2017;7(2):e013537. doi: 10.1136/bmjopen-2016-013537 [published Online First: 2017/02/19]

For peer review only - http://bmjopen.bmj.com/site/about/guidelines.xhtml

31. Rasmussen KM, Catalano PM, Yaktine AL. New guidelines for weight gain during pregnancy: what obstetrician/gynecologists should know. *Curr Opin Obstet Gynecol* 2009;21(6):521-6. doi: 10.1097/GCO.0b013e328332d24e [published Online First: 2009/10/08]
32. Simpson E, Bradley J, Poliakov I, et al. Iterative Development of an Online Dietary Recall Tool: INTAKE24. *Nutrients* 2017;9(2) doi: 10.3390/nu9020118 [published Online First: 2017/02/18]
33. Foster E DJ, Simpson E, et al. Comparison study: INTAKE24 vs Interviewer led recall. Final report, 2014.
34. EuroQoL. EQ-5D. Available from: https://euroqol.org/. Accessed March 2020.

For peer review only

For peer review only - http://bmjopen.bmj.com/site/about/guidelines.xhtml

Figure 1. Study flow. BMI: body mass index; OGTT: oral glucose tolerance test; GDM: gestational diabetes

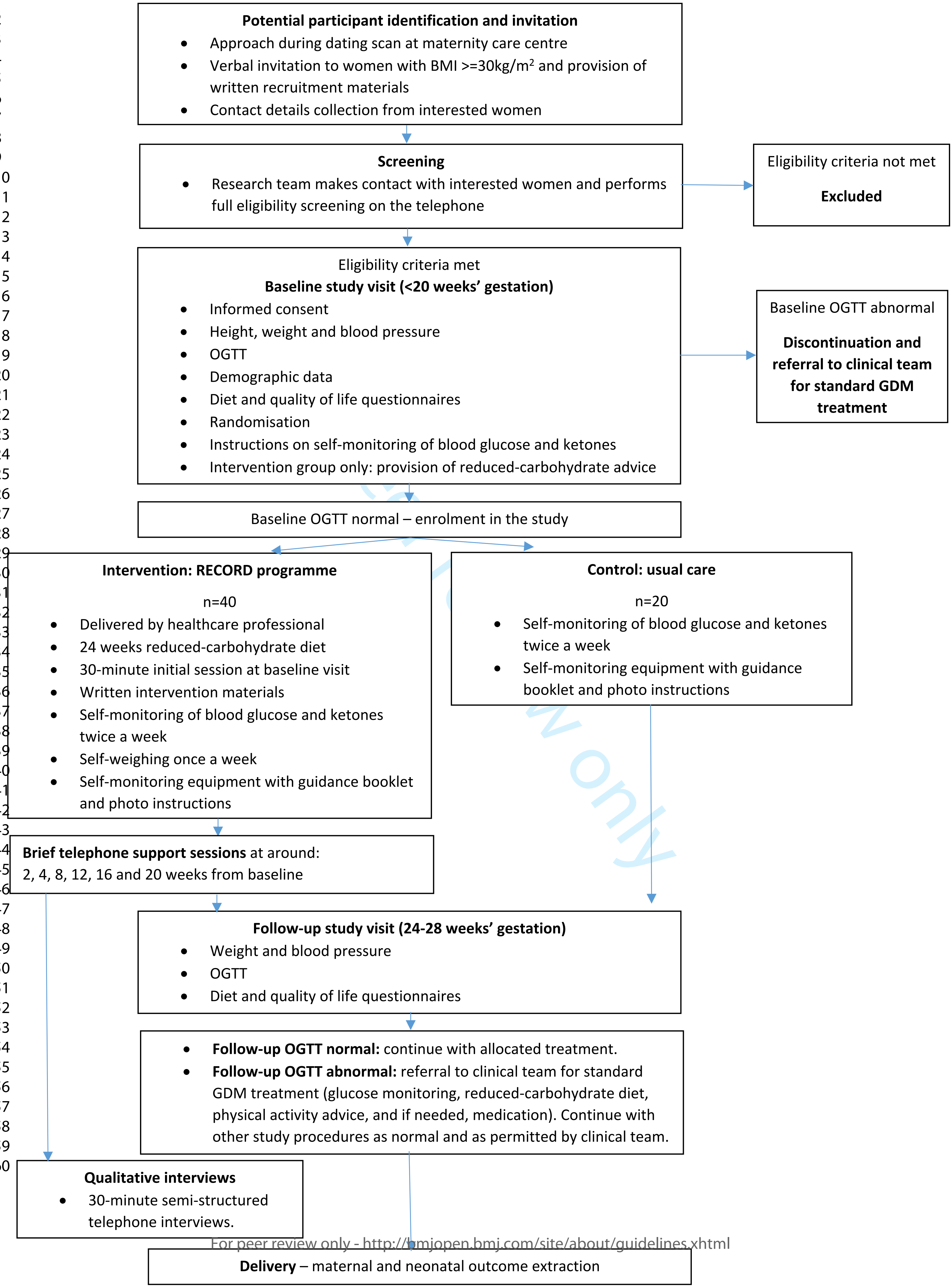

For peer review only

Figure 2. Progression criteria. OGTT: Oral Glucose Tolerance Test

**Criteria**	**How it will be assessed?**	**Indication of success**	
		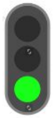	**95% Confidence Intervals that don't cross 170g total carbohydrate /day**
**Adoption of the intervention**	**Total carbohydrate consumption via 24-hour recall online diet questionnaire at baseline and follow-up.**	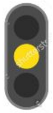	**95% Confidence Intervals that cross both 140g and 170g total carbohydrate/day**
		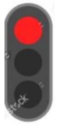	**95% Confidence Intervals that don't include 140g or 170g total carbohydrate/day**
		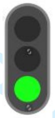	**95% Confidence Intervals that don't cross 70%**
**Retention rate**	**Proportion of all enrolled participants who complete an OGTT at follow-up (24-28 weeks' gestation).**	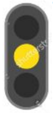	**95% Confidence Intervals that cross both 70% and 80%**
		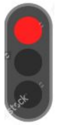	**95% Confidence Intervals that don't include 70% or 80%**

For peer review only

SPIRIT

STANDARD PROTOCOL ITEMS: RECOMMENDATIONS FOR INTERVENTIONAL TRIALS

SPIRIT 2013 Checklist: Recommended items to address in a clinical trial protocol and related documents*

Section/item	ItemNo	Description	Lines
**Administrative information**			
Title	1	Descriptive title identifying the study design, population, interventions, and, if applicable, trial acronym	1-3
Trial registration	2a	Trial identifier and registry name. If not yet registered, name of intended registry	40
	2b	All items from the World Health Organization Trial Registration Data Set	40
Protocol version	3	Date and version identifier	433
Funding	4	Sources and types of financial, material, and other support	442-443, 445-447
Roles and responsibilities	5a	Names, affiliations, and roles of protocol contributors	7-12
	5b	Name and contact information for the trial sponsor	442-443
	5c	Role of study sponsor and funders, if any, in study design; collection, management, analysis, and interpretation of data; writing of the report; and the decision to submit the report for publication, including whether they will have ultimate authority over any of these activities	449-452
	5d	Composition, roles, and responsibilities of the coordinating centre, steering committee, endpoint adjudication committee, data management team, and other individuals or groups overseeing the trial, if applicable (see Item 21a for data monitoring committee)	405-410

For peer review only

**Introduction**			
Background and rationale	6a	Description of research question and justification for undertaking the trial, including summary of relevant studies (published and unpublished) examining benefits and harms for each intervention	58-112
	6b	Explanation for choice of comparators	N/A (usual care)
Objectives	7	Specific objectives or hypotheses	106-112
Trial design	8	Description of trial design including type of trial (eg, parallel group, crossover, factorial, single group), allocation ratio, and framework (eg, superiority, equivalence, noninferiority, exploratory)	Line 107 and Methods: 113-118
**Methods: Participants, interventions, and outcomes**			
Study setting	9	Description of study settings (eg, community clinic, academic hospital) and list of countries where data will be collected. Reference to where list of study sites can be obtained	120
Eligibility criteria	10	Inclusion and exclusion criteria for participants. If applicable, eligibility criteria for study centres and individuals who will perform the interventions (eg, surgeons, psychotherapists)	130-157
Interventions	11a	Interventions for each group with sufficient detail to allow replication, including how and when they will be administered	191-254
	11b	Criteria for discontinuing or modifying allocated interventions for a given trial participant (eg, drug dose change in response to harms, participant request, or improving/worsening disease)	361-366
	11c	Strategies to improve adherence to intervention protocols, and any procedures for monitoring adherence (eg, drug tablet return, laboratory tests)	352-360

For peer review only - http://bmjopen.bmj.com/site/about/guidelines.xhtml

	11d	Relevant concomitant care and interventions that are permitted or prohibited during the trial	251-252, 367-378
Outcomes	12	Primary, secondary, and other outcomes, including the specific measurement variable (eg, systolic blood pressure), analysis metric (eg, change from baseline, final value, time to event), method of aggregation (eg, median, proportion), and time point for each outcome. Explanation of the clinical relevance of chosen efficacy and harm outcomes is strongly recommended	268-315
Participant timeline	13	Time schedule of enrolment, interventions (including any run-ins and washouts), assessments, and visits for participants. A schematic diagram is highly recommended (see Figure)	Figure 1 and Table 1
Sample size	14	Estimated number of participants needed to achieve study objectives and how it was determined, including clinical and statistical assumptions supporting any sample size calculations	172-183
Recruitment	15	Strategies for achieving adequate participant enrolment to reach target sample size	119-129
**Methods: Assignment of interventions (for controlled trials)**			
Allocation:			
Sequence generation	16a	Method of generating the allocation sequence (eg, computer-generated random numbers), and list of any factors for stratification. To reduce predictability of a random sequence, details of any planned restriction (eg, blocking) should be provided in a separate document that is unavailable to those who enrol participants or assign interventions	184-190
Allocation concealment mechanism	16b	Mechanism of implementing the allocation sequence (eg, central telephone; sequentially numbered, opaque, sealed envelopes), describing any steps to conceal the sequence until interventions are assigned	184-190
Implementation	16c	Who will generate the allocation sequence, who will enrol participants, and who will assign participants to interventions	184-190

Blinding (masking)	17a	Who will be blinded after assignment to interventions (eg, trial participants, care providers, outcome assessors, data analysts), and how	188-190
	17b	If blinded, circumstances under which unblinding is permissible, and procedure for revealing a participant's allocated intervention during the trial	N/A
**Methods: Data collection, management, and analysis**			
Data collection methods	18a	Plans for assessment and collection of outcome, baseline, and other trial data, including any related processes to promote data quality (eg, duplicate measurements, training of assessors) and a description of study instruments (eg, questionnaires, laboratory tests) along with their reliability and validity, if known. Reference to where data collection forms can be found, if not in the protocol	316-350
	18b	Plans to promote participant retention and complete follow-up, including list of any outcome data to be collected for participants who discontinue or deviate from intervention protocols	352-360
Data management	19	Plans for data entry, coding, security, and storage, including any related processes to promote data quality (eg, double data entry; range checks for data values). Reference to where details of data management procedures can be found, if not in the protocol	415-431
Statistical methods	20a	Statistical methods for analysing primary and secondary outcomes. Reference to where other details of the statistical analysis plan can be found, if not in the protocol	379-391
	20b	Methods for any additional analyses (eg, subgroup and adjusted analyses)	379-391
	20c	Definition of analysis population relating to protocol non-adherence (eg, as randomised analysis), and any statistical methods to handle missing data (eg, multiple imputation)	379-391
**Methods: Monitoring**			

For peer review only - http://bmjopen.bmj.com/site/about/guidelines.xhtml

Data monitoring	21a	Composition of data monitoring committee (DMC); summary of its role and reporting structure; statement of whether it is independent from the sponsor and competing interests; and reference to where further details about its charter can be found, if not in the protocol. Alternatively, an explanation of why a DMC is not needed	405-410
	21b	Description of any interim analyses and stopping guidelines, including who will have access to these interim results and make the final decision to terminate the trial	405-410
Harms	22	Plans for collecting, assessing, reporting, and managing solicited and spontaneously reported adverse events and other unintended effects of trial interventions or trial conduct	411-414
Auditing	23	Frequency and procedures for auditing trial conduct, if any, and whether the process will be independent from investigators and the sponsor	405-410
**Ethics and dissemination**			
Research ethics approval	24	Plans for seeking research ethics committee/institutional review board (REC/IRB) approval	432-441
Protocol amendments	25	Plans for communicating important protocol modifications (eg, changes to eligibility criteria, outcomes, analyses) to relevant parties (eg, investigators, REC/IRBs, trial participants, trial registries, journals, regulators)	436-438
Consent or assent	26a	Who will obtain informed consent or assent from potential trial participants or authorised surrogates, and how (see Item 32)	159-161
	26b	Additional consent provisions for collection and use of participant data and biological specimens in ancillary studies, if applicable	N/A
Confidentiality	27	How personal information about potential and enrolled participants will be collected, shared, and maintained in order to protect confidentiality before, during, and after the trial	415-431
Declaration of interests	28	Financial and other competing interests for principal investigators for the overall trial and each study site	463-464

For peer review only - http://bmjopen.bmj.com/site/about/guidelines.xhtml

Access to data	29	Statement of who will have access to the final trial dataset, and disclosure of contractual agreements that limit such access for investigators	428-431
Ancillary and post-trial care	30	Provisions, if any, for ancillary and post-trial care, and for compensation to those who suffer harm from trial participation	Post-trial care N/A<br><br>Compensation: Sponsor
Dissemination policy	31a	Plans for investigators and sponsor to communicate trial results to participants, healthcare professionals, the public, and other relevant groups (eg, via publication, reporting in results databases, or other data sharing arrangements), including any publication restrictions	438-441
	31b	Authorship eligibility guidelines and any intended use of professional writers	N/A
	31c	Plans, if any, for granting public access to the full protocol, participant-level dataset, and statistical code	438-441
**Appendices**			
Informed consent materials	32	Model consent form and other related documentation given to participants and authorised surrogates	Attached
Biological specimens	33	Plans for collection, laboratory evaluation, and storage of biological specimens for genetic or molecular analysis in the current trial and for future use in ancillary studies, if applicable	N/A, as per NHS procedures

*It is strongly recommended that this checklist be read in conjunction with the SPIRIT 2013 Explanation & Elaboration for important clarification on the items. Amendments to the protocol should be tracked and dated. The SPIRIT checklist is copyrighted by the SPIRIT Group under the Creative Commons "Attribution-NonCommercial-NoDerivs 3.0 Unported" license.

For peer review only - http://bmjopen.bmj.com/site/about/guidelines.xhtml

NUFFIELD DEPARTMENT OF PRIMARY CARE HEALTH SCIENCES

UNIVERSITY OF OXFORD

RECORD

Reduced-carbohydrate intervention to prevent gestational diabetes

Chief Investigator:
Dr Nerys Astbury
DPhil Student:
Moscho Michalopoulou
Tel: 01865 617 857
Email: record@phc.ox.ac.uk

RECORD

Reduced-carbohydrate intervention to prevent gestational diabetes

PARTICIPANT INFORMATION SHEET

We would like to invite you to take part in this research study. Before you decide, it is important that you understand why the research is being done and what it would involve for you. Please take time to read this information, and discuss it with others if you wish. If anything is not clear, or if you would like more information, please ask us.

What is the purpose of the study?

This study is a PhD educational project, aiming to test if it is possible to deliver a dietary programme designed to reduce the risk that women who are pregnant will develop gestational diabetes (GDM). GDM is a condition that develops usually during the second or third trimester of pregnancy, in which women's blood glucose levels are high. It can lead to health complications for the mother and the baby during pregnancy and later in life. Carrying or gaining too much weight during pregnancy, increases the chances of developing GDM. Effective ways to help women who are at increased risk of GDM to control their weight and blood glucose levels early in pregnancy are needed.

What we eat affects our weight and blood glucose levels. Carbohydrates are the main source of energy (calories) and eating carbohydrates leads to rise in blood glucose. We therefore developed a new dietary programme to help you moderately reduce, but not cut out, the amount of carbohydrate that you eat, while maintaining a balanced diet.

This small study will test if it is possible to deliver the new programme alongside routine appointments and how well women can follow this advice for approximately 6 months. We will monitor the impact of the programme on some markers in the blood, and explore its effect on weight gain and other health outcomes throughout pregnancy. If the programme is feasible and well-liked, we will run a bigger study aiming to help prevent GDM.

Why have I been invited?

We are looking for 60 women who are at increased risk of developing GDM. Your most recent weight and height measurements suggest that you may have a raised Body Mass Index, which is a risk factor for GDM.

Do I have to take part?

No, you don't have to take part. Taking part is voluntary. If you decide not to take part, this will not affect the care you receive during pregnancy and delivery. If you decide to take part but then change your mind, you can withdraw at any time without giving us a reason. This will not affect the care that you receive.

What will happen to me if I decide to take part?

If you decide to take part, you will be asked to attend two research study visits at the hospital with the study team. One visit will happen before you are 20 weeks pregnant, and the other visit will happen around 24-28 weeks of pregnancy. This second visit replaces a visit that you would be required to attend even if you were not taking part in the study. Each visit will be scheduled in the morning and will last for approximately 2 hours.

Preparation for study visits: You will be asked to arrive to these appointments after an overnight fast. This means you should not eat or drink anything (apart from water) from the night before the visit. You should also avoid smoking, vaping, using nicotine replacement therapy and chewing sugar gum since the previous night.

First study visit: At the first study visit (before 20 weeks of pregnancy), the researcher will go through this information sheet with you and you will have the opportunity to ask any questions about the study. If you are happy to take part, you will be asked to sign a consent form and you will be given a copy to keep. With your consent, we will complete the following procedures:

- **Oral Glucose Tolerance Test (OGTT):** We will collect a small blood sample and then ask you to drink a special sugar drink. We will collect further small blood samples after 1 and 2 hours. The OGTT tests how well your body handles the ingestion of sugar.
- **Study assessments:** Whilst you are waiting, we will measure your height, weight and blood pressure, and ask you to fill in questionnaires about yourself, including your usual diet and quality of life.

For peer review only - http://bmjopen.bmj.com/site/about/guidelines.xhtml

You will also be allocated to one of the study groups. Two-thirds of participants will be assigned to the RECORD diet group, who will be guided to moderately reduce their carbohydrate intake, whilst the other participants will be assigned to the control group, who will continue to receive the usual care and support given to women who are pregnant. Having a control group helps us test if our intervention is more beneficial compared to the usual dietary advice. Group allocation is entirely down to chance and will be decided by a computer programme. You cannot choose which group you are in and you won't be able to change group allocation once you have been allocated.

Two groups:

- **Intervention group:** If you are assigned to the RECORD diet group, we ask that you follow the dietary advice and recommendations to the best of your ability until the delivery of your baby. During the first study visit, you will be advised to moderately reduce, but not cut out, the amount of carbohydrate in your diet. You will receive a booklet, meal and recipe ideas and other materials to help you follow the dietary advice. We will arrange another six brief sessions to support you through the diet plan, at a convenient time, when you are around 16, 18, 20, 24-28, 30 and 34 weeks pregnant. These sessions will be by telephone, except the one at 24-28 weeks, which is on the same day as your second study visit and can be either on the telephone or face-to-face. All support sessions will take a maximum of 15 minutes. We will ask your permission to audio-record the face-to-face and telephone sessions. You will also be asked to monitor your weight once a week and send the measurements to the study team. We can lend you weighing scales if you don't own a set.
- **Control group:** If you're assigned to the control group, you will continue to receive any dietary advice that is given as part of the routine pregnancy care.

Blood glucose and ketone monitoring: Everyone taking part will be asked to measure their blood glucose and ketone levels at home twice a week for the duration of the study, using a monitor we will provide. You can log your measurements into a mobile phone/tablet/iPad application or text them to the study team. The study team and members of the clinical care team will have access to these readings. Taking part in this study will not affect any other treatment you may be receiving.

Follow-up study visit: During the follow-up appointment at 24-28 weeks of pregnancy, we will repeat the procedures conducted at the first study visit. The OGTT conducted at this visit will determine whether you have developed GDM. You would usually have this same test at this time at the hospital or your GP practice, even if you were not taking part in the study. To avoid having the same test twice, our test will replace the test you would usually have and we will share the results with your clinical care team.

Diagnosis of GDM: If the results of this second OGTT indicate that you have developed GDM, you will have the same access to medical care and any necessary medications, regardless of your study group allocation. Everyone will be offered routine care dietary advice for GDM, which is similar to that we are testing in this study. Therefore, if you are in the intervention group, you can continue following the RECORD moderately reduced-carbohydrate programme, and weigh yourself weekly until delivery.

Optional discussion: After the follow-up visit, if you are in the RECORD diet group, we may ask if you would be willing to have an in-depth discussion on the phone with a member of the study team about your experiences of the intervention. This is entirely optional. We will provide you with more details during your follow-up visit.

End of study-health outcomes: With your consent, we will access your medical records after the delivery of your baby. This will enable us to compare health outcomes of the mothers and new-borns between the two study groups.

What should I consider?

You may not be able to take part if you have certain medical conditions or taking medication that might make the dietary advice we are giving unsuitable for you. These include: taking metformin, being diagnosed with GDM at the first study visit, having diabetes before pregnancy, a serious medical condition (e.g. heart failure, serious liver or kidney disease, or disease of the nervous system), serious mental health problems requiring hospital stays, current or previous eating disorders affecting your physical and mental health, or very bad nausea or vomiting. To be able to take part, we also need to confirm that you are not carrying a baby with a serious medical condition identified during scans, that you have not had an organ transplant or weight loss surgery, that you are able to understand spoken and written English, and that you are not taking part in other intervention research studies that may affect your weight, health behaviours or blood glucose control. If you are eligible and decide to take part, you can continue to take your regular prescribed or over-the-counter medication during the study.

For peer review only - http://bmjopen.bmj.com/site/about/guidelines.xhtml

Are there any possible disadvantages or risks from taking part?

Severely restricting carbohydrate intake may lead to increased ketone levels, compounds that are produced when glucose – which mainly comes from carbohydrate foods – is not available to the body. It is not expected that the RECORD programme will lead to increased ketones because it has been designed to ensure you get adequate amount of carbohydrate which is the same as advised in routine care if women are diagnosed with GDM. The only difference is that we ask you to follow this advice earlier in pregnancy and in a more structured way. In addition, no evidence is available to determine any effect of the diet on the unborn child. We will provide you with a device to monitor both your blood glucose and ketone levels as an extra check. We will also ask you to measure your blood glucose and ketones if you are unwell and contact your clinical care team for advice if required.

You will be asked to fast overnight before each study visit. Some people can feel lightheaded or unwell if they don't eat for long periods of time. Once we have finished the tests, we will provide you with a light breakfast before you leave the hospital. You will be asked to provide small blood samples and as with any blood sample, there is possibility of stinging and a risk that you develop some bruising around the area, which should go away in a few days. Some people may feel faint while the sample is taken, but all blood samples will be taken by health care professionals who are trained and experienced in these procedures. Finally, all measures against coronavirus will be taken during the study visits as per Government guidance.

What are the possible benefits of taking part?

If you are randomised to the RECORD diet group you will receive detailed advice, support and guidance on your diet early in pregnancy. In addition, the knowledge gained in this study may help to plan a bigger study which may help in the future other women who are pregnant, to control their weight gain and blood glucose levels with diet alone, and to possibly reduce their risk of developing GDM.

Will my General Practitioner/family doctor (GP) be informed of my participation?

With your consent, we will notify your GP of your participation in this study and we will share with them the results of any relevant blood tests.

Will my taking part in the study be kept confidential?

Any data that we collect about you during this study will be kept strictly confidential. You will be assigned a unique study code to avoid identification with your name. Responsible members of the University of Oxford and the Oxford University Hospitals NHS Foundation Trust, may be given access to data for monitoring and/or audit of the study to ensure that the research is complying with applicable regulations.

Will I be reimbursed for taking part?

You will be offered £30 at each study visit (£60 in total) in the form of gift cards or bank transfer according to your preference, to compensate you for the time, inconvenience and expenses such as parking costs, associated with attending the study visits. If you prefer a bank transfer, we will ask your permission to collect your bank details.

What will happen to the samples I give?

Your blood samples will be analysed to measure blood glucose, HbA1c (average blood glucose levels of the past 2-3 months), insulin, and to estimate a measure of insulin resistance. The samples will then be destroyed and will not be used for other purposes.

What will happen to my data?

Data protection regulation requires that we state the legal basis for processing information about you. In the case of research, this is 'a task in the public interest.' The University of Oxford is the data controller and is responsible for looking after your information and using it properly.

We will be using information from you and your medical records, in order to undertake this study and will use the minimum personally-identifiable information possible. We will keep identifiable information about you for a year after the study has finished (e.g. contact details), in order to prepare and send you a summary of the study results. This excludes any research documents with personal information, such as consent forms, which will be held securely at the University of Oxford for 5 years after the end of the study. Audio-recordings from the intervention sessions will be encrypted and kept securely in a password protected computer at the University of Oxford. Members of the study team will listen to these audio-recordings to assess how well the intervention is delivered. The audio files will then be deleted. The Oxford University Hospitals NHS Foundation Trust will use your name, home address and

For peer review only - http://bmjopen.bmj.com/site/about/guidelines.xhtml

contact details, to contact you about the research study, and to oversee the quality of the study. They will keep identifiable information about you from this study in keeping with local policies for retention of medical records. Data protection regulation provides you with control over your personal data and how it is used. When you agree to your information being used in research, however, some of those rights may be limited in order for the research to be reliable and accurate. Further information about your rights with respect to your personal data is available at https://compliance.web.ox.ac.uk/individual-rights .To find out more about how we use your information contact us on 01865 617 857 or record@phc.ox.ac.uk .

What will happen if I don't want to carry on with the study?

If you decide now that you want to take part, but change your mind later, you can withdraw from the study at any point and without giving us a reason. If you decide to withdraw we will use any data, including blood samples, which we have collected from you up to the point at which you decide to withdraw, unless you explicitly request us to delete/destroy them.

What will happen to the results of this study?

The results of this study will help us design future studies with the aim of helping women who are pregnant to reduce their risk of developing GDM. These studies may help to change guidelines about ways to prevent GDM. The overall study results may be presented at scientific conferences or published in scientific journals. This research will also contribute to the fulfilment of a doctoral thesis and educational qualification (Doctor of Philosophy) at the University of Oxford. On successful submission of the thesis, it will be deposited in the University archives, and will be available to the public. All results will be presented or published in a form that does not identify you. We will send you a summary of the study results once the study has finished and data has been analysed (from late 2022 onward).

What if we find something unexpected?

We will share any relevant blood test results with your direct clinical care team and GP. They will be in touch if anything unexpected is found.

What if there is a problem?

The University of Oxford, as Sponsor, has appropriate insurance in place in the unlikely event that you suffer any harm as a direct consequence of your participation in this study. NHS indemnity operates in respect of the clinical treatment which is provided. If you wish to complain about any aspect of the way in which you have been approached or treated, or how your information is handled during the course of this study, you should contact Dr Nerys Astbury or Moscho Michalopoulou on 01865 617 857 or record@phc.ox.ac.uk ; or you may contact the University of Oxford Clinical Trials and Research Governance (CTRG) office on 01865 616480, or the head of CTRG, email: ctrg@admin.ox.ac.uk .The Patient Advisory Liaison Service (PALS) is a confidential NHS service that can provide you with support for any complaints or queries you may have regarding the care you receive as an NHS patient. PALS is unable to provide information about this research study. If you wish to contact the PALS team please contact 01865 221473 or PALS@ouh.nhs.uk .

How have patients and the public been involved in this study?

We have discussed this research with women affected by GDM. They helped to shape the ideas for this research and to determine the questions we are trying to answer. In designing this study, we have taken into account their opinions on what information women who are pregnant would like to have available to help them change their diet in order to control their weight and blood glucose levels. A patient representative has provided feedback on the intervention materials and will continue to be involved throughout the study.

Who is organising and funding the study?

The study is sponsored by the University of Oxford. The research is funded by the Oxford-Medical Research Council Doctoral Training Partnership and the National Institute for Health Research Oxford Biomedical Research Centre.

Who has reviewed the study?

Research in the NHS is looked at by an independent group of people, called a Research Ethics Committee, to protect participants' interests. The South-Central Oxford B Research Ethics Committee has approved the ethics of this study.

Further information and contact details

Please contact Dr Nerys Astbury or Moscho Michalopoulou on 01865 617 857 or record@phc.ox.ac.uk .

Thank you for considering taking part in this study.

For peer review only - http://bmjopen.bmj.com/site/about/guidelines.xhtml

NUFFIELD DEPARTMENT OF PRIMARY CARE HEALTH SCIENCES

UNIVERSITY OF OXFORD

RECORD

Reduced-carbohydrate intervention to prevent gestational diabetes

Chief Investigator: Dr Nerys Astbury
DPhil Student: Moscho Michalopoulou
Tel: 01865 617 857
Email: record@phc.ox.ac.uk

RECORD

Reduced-carbohydrate intervention to prevent gestational diabetes-Qualitative Interviews

PARTICIPANT INFORMATION SHEET

We would like to invite you to take part in an informal interview about your experiences of the RECORD study. Before you decide, it is important that you understand why this interview is being done and what it would involve for you. Please take time to read this information, and discuss it with others if you wish. If there is anything that is not clear, or if you would like more information, please ask us.

What is the purpose of the informal interview?

We would like to know how participants feel about the RECORD intervention. We will discuss with you to understand your experiences of the dietary programme and use the feedback to refine the intervention.

Why have I been invited?

We are looking for up to 30 women from the main RECORD study so that we can explore their views about the RECORD diet advice. You have been invited to take part because you have been allocated to the RECORD diet intervention group.

Do I have to take part?

No, taking part in the interview is entirely voluntary. You may decide not to take part in the interview and this will not affect your participation in the main RECORD study or the clinical care you receive. If you decide now to take part, but at a later time decide that you no longer wish to participate, you can withdraw from the study. If you decide to withdraw for any reason, we will keep any information already collected about you.

What will happen to me if I decide to take part?

We will invite you to talk about your experience of the RECORD intervention and provide feedback, on a day and time that is convenient for you. This will involve discussing with the researcher over the phone for approximately 30 minutes. We will audio-record this discussion.

What should I consider?

We would expect you to talk about your experiences of attempting to follow the RECORD intervention, of taking part in the study, and of monitoring your weight, blood glucose and ketone levels. The discussion will be audio-recorded.

Are there any possible disadvantages or risks from taking part?

The risks of taking part in this interview are extremely minimal. However, it is possible that some of the questions asked during the interview may cause you embarrassment or distress. The questions will focus on exploring your experiences of the dietary advice you were given and of taking part in the main RECORD study. If you feel uncomfortable about answering any of the questions, you can decline to answer any question and the interviewer will move on to the next question.

What are the possible benefits of taking part?

Although there are no direct benefits to you from taking part, your participation may help researchers understand which components of the dietary intervention work well and which don't work so well. This may help future decisions with respect to making consistent dietary advice routinely available during antenatal care in the NHS.

For peer review only - http://bmjopen.bmj.com/site/about/guidelines.xhtml

Will my taking part in the informal interview be kept confidential?

Any information that we collect about you during this discussion will be kept strictly confidential. We will not disclose your participation in these interviews to your GP. We will use a unique study code to avoid identification with your name. Responsible members of the University of Oxford and the Oxford University Hospitals NHS Foundation Trust, may be given access to data for monitoring and/or audit of the study to ensure that the research is complying with applicable regulations.

Will I be reimbursed for taking part?

You will be compensated for your time with £20 for taking part in the interview in the form of a gift card or bank transfer, according to your preference. This is in addition to any compensation you may receive from taking part in the main RECORD study. If you prefer a bank transfer, we will ask your permission to collect your bank details (if not already done so).

What will happen to my data?

Data protection regulation requires that we state the legal basis for processing information about you. In the case of research, this is 'a task in the public interest.' The University of Oxford is the data controller and is responsible for looking after your information and using it properly.

We will use your contact details to arrange the interview and will use the minimum personally-identifiable information possible. We will keep identifiable information for a year after the main study has finished (e.g. contact details), in order to prepare and send you a summary of the study results. We will ask you to verbally consent on the telephone to take part in the interviews and will record your consent on a consent form. We will send a signed copy of the consent form to you in the post. The interviews will be audio-recorded. The audio-recording will be encrypted and stored securely on a password protected server separately from any personal information held about you. The audio-recording will then be typed-out by an approved transcriber service. The recording passed to the transcriber will be identified by a study code number, rather than any of your personal details. The transcriber will not retain the recording after they have been typed out and returned to the research team. This processing of data will be formalised in a written agreement to ensure it is undertaken confidentially and securely. The original audio-recording will then be deleted. The original consent form with your recorded verbal consent will be held securely at the University of Oxford for 5 years after the end of the main RECORD study and will then be destroyed. The pseudonymised transcription from your audio-recorded interview will be stored securely on an encrypted and password-protected server for 3 years after the end of the main study and will then be deleted.

Data protection regulation provides you with control over your personal data and how it is used. When you agree to your information being used in research, however, some of those rights may be limited in order for the research to be reliable and accurate. Further information about your rights with respect to your personal data is available at https://compliance.web.ox.ac.uk/individual-rights .You can find out more about how we use your information by contacting us on 01865 617 857 or record@phc.ox.ac.uk .

What will happen if I don't want to carry on with the interview?

Participation is voluntary and participants may change their minds at a later stage. If you decide to withdraw from this interview, it will not affect the care you receive from any relevant service. If you decide to withdraw from this interview, you will remain in the main RECORD study, unless you notify us otherwise. If you withdraw from this interview after we have already started the discussion, unless you state otherwise, any data already collected will be used as detailed in this participant information sheet.

What will happen to the results of this study?

The transcriptions of the interviews will be coded and all these coded interviews will be analysed to determine whether there are similar themes identified. We plan to publish the findings in scientific journals and present the results at conferences. Please be reassured that you will not be identified in these outputs. If your quotes are used

For peer review only - http://bmjopen.bmj.com/site/about/guidelines.xhtml

they will be identified by an anonymous pseudonym. When these publications or reports become available (from late 2022 onward), we will send you a copy of the results. Some of the research being undertaken will also contribute to the fulfilment of a doctoral thesis and educational qualification (Doctor of Philosophy) at the University of Oxford.

What if there is a problem?

The University of Oxford, as Sponsor, has appropriate insurance in place in the unlikely event that you suffer any harm as a direct consequence of your participation in this interview. If you wish to complain about any aspect of the way in which you have been approached or treated during the course of this study, you should contact the Chief Investigator Dr Nerys Astbury, or the DPhil Student and main researcher, Moscho Michalopoulou, on 01865 617 857 or record@phc.ox.ac.uk . Alternatively, you may contact the University of Oxford Clinical Trials and Research Governance (CTRG) office on 01865 616480, or ctrg@admin.ox.ac.uk .

How have patients and the public been involved in this study?

Potential participants have been actively involved in developing the questions asked during the interviews.

Who is organising and funding the study?

The RECORD study, including the informal interviews, is sponsored by the University of Oxford. The research is funded by the Oxford-Medical Research Council (MRC) Doctoral Training Partnership and the National Institute for Health Research (NIHR) Oxford Biomedical Research Centre.

Who has reviewed the study?

All research in the NHS is looked at by an independent group of people, called a Research Ethics Committee, to protect participants' interests. The South-Central Oxford B Research Ethics Committee has approved the ethics of this study.

Further information and contact details:

Please contact Dr Nerys Astbury or Moscho Michalopoulou on 01865 617 857 or record@phc.ox.ac.uk .

Thank you for reading this information.

For peer review only - http://bmjopen.bmj.com/site/about/guidelines.xhtml

NUFFIELD DEPARTMENT OF PRIMARY CARE HEALTH SCIENCES
UNIVERSITY OF OXFORD

RECORD
Reduced-carbohydrate intervention to prevent gestational diabetes

Chief Investigator: Dr Nerys Astbury
DPhil Student: Moscho Michalopoulou
Tel: 01865 617 857
Email: record@phc.ox.ac.uk

FLOWCHART

Reduced-carbohydrate intervention to prevent gestational diabetes

First study visit
2 hours
(all women)

Before 20 weeks of pregnancy:

- Eligibility assessment.
- Informed consent.
- Oral Glucose Tolerance Test (OGTT): fasting blood sample → sugar drink → blood samples after 1 and 2 hours
- Height, weight and blood pressure measurements.
- Questionnaires about yourself, including usual diet and quality of life.
- Random allocation to one of the two study groups: reduced-carbohydrate dietary programme (group 1) or usual care (group 2).
- Advice to both groups on monitoring blood glucose and ketones until giving birth.
- Group 1 only: dietary advice, pack with resources, advice on weight monitoring.

Intervention period
(group 1 only)

- Engagement with the diet intervention and weight monitoring until giving birth.
- Extra 15-minute telephone sessions for support, at around 16, 18, 20, 24-28 (can also be done face-to-face during the second study visit if preferred), 30 and 34 weeks of pregnancy.

Second study visit
2 hours – follow-up
(all participants)

At around 24-28 weeks of pregnancy:

- Second OGTT to detect if you have developed gestational diabetes.
- Measurement of weight and blood pressure.
- Questionnaires about diet and quality of life.

Optional discussion
(group 1 only)

- 30-minute telephone discussion about your experiences of the intervention (we will inform you more about this during the second study visit)

Flowchart of Participation	Version 1.2, 12th July 2021
RECORD study	IRAS Project number: 279623
Chief Investigator: Dr Nerys Astbury	REC Reference number: 20/SC/0442
	Page 1 of 1

For peer review only

Development and testing of a reduced carbohydrate intervention for the management of obesity and reduction of gestational diabetes (RECORD): protocol for a feasibility randomised controlled trial

Journal:	*BMJ Open*
Manuscript ID	bmjopen-2022-060951.R1
Article Type:	Protocol
Date Submitted by the Author:	02-Jul-2022
Complete List of Authors:	Michalopoulou, Moscho; University of Oxford, Nuffield Department of Primary Care Health Sciences; NIHR Oxford Biomedical Research Centre<br>Jebb, Susan; University of Oxford, Nuffield Department of Primary Care Health Sciences; NIHR Oxford Biomedical Research Centre<br>MacKillop, Lucy; University of Oxford Nuffield Department of Women's & Reproductive Health; Oxford University Hospitals NHS Foundation Trust<br>Dyson, Pamela; University of Oxford, Oxford Centre for Diabetes, Endocrinology and Metabolism (OCDEM), ; NIHR Oxford Biomedical Research Centre<br>Hirst, Jane E; University of Oxford, Nuffield Department of Women's and Reproductive Health; Oxford University Hospitals NHS Foundation Trust<br>Wire, Amy; Berkshire Healthcare NHS Foundation Trust<br>Astbury, Nerys; University of Oxford, Nuffiled Department of Primary Care Health Sciences; NIHR Oxford Biomedical Research Centre
**Primary Subject Heading**:	Nutrition and metabolism
Secondary Subject Heading:	Diabetes and endocrinology, Obstetrics and gynaecology
Keywords:	Diabetes in pregnancy < DIABETES & ENDOCRINOLOGY, NUTRITION & DIETETICS, OBSTETRICS

SCHOLARONE™
Manuscripts

Development and testing of a reduced carbohydrate intervention for the management of obesity and reduction of gestational diabetes (RECORD): protocol for a feasibility randomised controlled trial

Moscho Michalopoulou,[1,5] Susan A Jebb,[1,5] Lucy Mackillop,[2,3] Pamela Dyson,[4,5] Jane E Hirst,[2,3] Amy Wire,[6] Nerys M Astbury[1,5]

[1] Nuffield Department of Primary Care Health Sciences, University of Oxford, United Kingdom

[2] Nuffield Department of Women's and Reproductive Health, University of Oxford, United Kingdom

[3] Oxford University Hospitals NHS Foundation Trust, United Kingdom

[4] Oxford Centre for Diabetes Endocrinology & Metabolism (OCDEM), United Kingdom

[5] National Institute for Health Research (NIHR) Oxford Biomedical Research Centre, United Kingdom

[6] Patient Representative

Corresponding Author: Moscho Michalopoulou, Nuffield Department of Primary Care Health Sciences, Radcliffe Primary Care Building, Woodstock Road, Oxford, OX2 6GG, email: moscho.michalopoulou@phc.ox.ac.uk

Word count: 5170

For peer review only

Abstract

Introduction:

Previous trials of dietary interventions to prevent gestational diabetes mellitus (GDM) have yielded only limited success. Low-carbohydrate diets have shown promise for the treatment of type 2 diabetes, but there is no evidence to support their use in pregnancy. The aim of this study is to explore the feasibility of a moderately reduced-carbohydrate dietary intervention delivered from mid-pregnancy alongside routine antenatal care.

Methods and analysis:

This is a feasibility randomised controlled trial (RCT) with embedded qualitative study. Sixty women who are pregnant <20 weeks' gestation, with body mass index ≥30 kg/m^2 at their antenatal booking appointment, will be randomised 2:1 intervention or control (usual care) and followed-up until delivery. The intervention is a moderately reduced-carbohydrate diet (~130-150g total carbohydrate/day), designed to be delivered alongside routine antenatal appointments. Primary outcomes are measures of adoption of the diet and retention of participants. Secondary outcomes include incidence of GDM, change in markers of glycaemic control, gestational weight gain, total carbohydrate and energy intake. Process outcomes will examine resources and management issues. Exploratory outcomes include further dietary changes, quality of life, maternal and neonatal outcomes, and qualitative measures.

Ethics and dissemination:

This trial was reviewed and approved by the South-Central Oxford B Research Ethics Committee NHS National Research Ethics Committee and the Health Research Authority (Reference: 20/SC/0442). The study results will inform whether to progress to a full-scale RCT to test the clinical effectiveness of the RECORD programme to prevent GDM in women at high risk. The findings will be published in peer reviewed journals and presented at conferences.

Trial registration: ISRCTN16235884

For peer review only

Strengths and limitations of this study

- The intervention has been co-developed by a multidisciplinary team of academic researchers and clinical professionals working in maternity, together with patients, to create a support programme likely to be acceptable to women and suitable for use in routine antenatal care.
- Quantitative and qualitative analyses will assess the feasibility (adherence and retention rate) and suitability of reducing carbohydrate intake in mid-pregnancy, to inform any progression to a future full trial.
- Dietary intake will be assessed at only two time points, and we will not be able to assess fluctuations in intake over time.
- This study is not powered to detect clinical effectiveness of the dietary intervention, and subsequent full-scale trials will be required to assess whether this intervention can effectively attenuate gestational weight gain and improve glycaemic control early in pregnancy, with the prospect of preventing gestational diabetes.

For peer review only

For peer review only - http://bmjopen.bmj.com/site/about/guidelines.xhtml

Introduction

Gestational diabetes mellitus (GDM), is defined as hyperglycaemia that develops or is first diagnosed during pregnancy.[1] GDM significantly increases the risk for adverse health outcomes for both the mother and the offspring,[2], [3-9] In the UK, approximately 16% of women who are pregnant develop GDM, but the prevalence reaches up to 25-30% in women living with obesity.[10] Excessive gestational weight gain (GWG) has also been proposed as a risk factor for GDM,[11] and several clinical trials in women with overweight or obesity during pregnancy, have tested whether interventions aimed at limiting GWG could help prevent GDM.[12-24] Although some trials have been successful in attenuating GWG, the majority have failed to demonstrate a reduced risk of developing GDM.[25], [26]

The small number of trials that have shown effectiveness in GDM prevention so far included intensive face-to-face dietetic support and/or provision of key foods to study participants, with no indication of how these strategies could be delivered in routine practice.[14], [16], [18], [22], [23] For example, the RADIEL study, reduced the incidence of GDM by 39% in women at high risk, but required extra face-to-face visits and a group session to deliver standard healthy eating and physical activity advice, which may not be manageable in routine practice given the amount of appointments women are already required to attend.[14] The ESTEEM and St Carlos trials, implemented a Mediterranean-style diet supplemented with extra virgin olive oil and nuts, provided as part of the trial, from early or mid-pregnancy, and showed a similar reduction in GDM risk.[16], [23] Nevertheless, wide scale implementation of a Mediterranean-style diet in the UK could be challenging due to limited understanding of the dietary pattern, differences in dietary habits and preferences, and the cost of extra virgin olive oil.[27-29]

The mechanism behind the success of some dietary interventions over others in preventing GDM remains unclear. Implementation at earlier stages of pregnancy seems to be key, but the

For peer review only - http://bmjopen.bmj.com/site/about/guidelines.xhtml

optimum dietary composition is unknown. In the RADIEL study, there was only a small improvement reported in adherence to the diet recommendations in the intervention group compared to the control group.[14] In the Mediterranean dietary interventions, it was speculated that increased unsaturated fat and polyphenol consumption might have been beneficial with regards to insulin sensitivity and inflammation.[16], [23] However, a reduction in dietary carbohydrate as a natural result of higher fat and protein consumption, in conditions when energy intake remains similar, may have also played a role. Indeed, successful interventions to date included some advice for reducing sugar consumption and/or moderating starchy foods.

Low-carbohydrate diets are effective for the management of type 2 diabetes, especially over the short-term (3-6 months).[30] Reducing carbohydrate intake from earlier stages of pregnancy may help reduce hyperglycaemia, independent of effects on weight gain, and may also help attenuate GWG by reducing energy intake. Although low-carbohydrate diets (<130g/day),[31] are not recommended for women who are pregnant, due to safety concerns associated with ketosis, research in non-pregnant populations suggests that significant ketosis only occurs when carbohydrate intake is severely restricted to <50g/day, leaving considerable scope to adopt a reduced-carbohydrate diet.[31] The level of carbohydrate reduction should be considered carefully though, particularly given that pregnancy is a ketosis-prone state, where ketones may develop at higher carbohydrate intakes than in non-pregnant populations.[32] One previous randomised controlled trial specifically aimed at reducing total carbohydrate consumption as part of an intensive healthy eating intervention.[33] Despite a substantial significant effect on limiting GWG, the intervention did not reduce GDM incidence. This study was underpowered to detect difference in GDM risk, and it resulted in only a small reduction in carbohydrate intake compared to usual care (reduction of 4.8% of energy intake from carbohydrates, equivalent to about 30g of carbohydrate per day). Thus, the effect of a reduced-carbohydrate diet as a preventative strategy for GDM remains to be investigated.

For peer review only - http://bmjopen.bmj.com/site/about/guidelines.xhtml

We have developed a dietary approach based on moderate carbohydrate reduction suitable for use in pregnancy, combined with behavioural support. The reduced-carbohydrate intervention for the management of obesity and reduction of gestational diabetes (RECORD), also recommends consumption of fresh foods, plenty of vegetables, and moderate protein intake. It was designed with midwives and dietitians experienced in working with women who are pregnant, together with patients, and is designed to be delivered alongside routine antenatal appointments. The aim of this trial is to assess the feasibility of the intervention and whether it leads to a substantial reduction in total carbohydrate intake, defined as target mean intake of around 130-150g/day, i.e. reduction of around 80-100g/day compared to the national average,[34] for the purposes of this study.

Other objectives include:

a) to assess the study procedures to inform the design of a full randomised controlled trial;
b) to assess the potential effect of the reduced-carbohydrate behavioural intervention on markers of glycaemic control, incidence of GDM, GWG, dietary intake, quality of life, and maternal and neonatal outcomes;
c) to qualitatively analyse participant experiences of the intervention.

Study design

A two-arm parallel group individually randomised controlled trial (RCT), in women who are at <20 weeks' gestation with a BMI ≥30kg/m^2. Participation will last approximately 6 months per participant; from early in the second trimester (no more than 19 weeks plus 6 days), until delivery. Participants will attend two study visits, one at baseline (<20 weeks' gestation) and one for follow-up, at 24-28 weeks' gestation.

For peer review only

Recruitment

Participants will be recruited from one maternity care centre in Oxfordshire, UK. The study is expected to run from May 2021 to December 2022. Women attending the dating or nuchal ultrasound scan appointment (typically between 8 and 14 weeks' gestation) will be approached to take part in the study. Potentially eligible participants will be provided with the participant information sheet, and invited to give their contact details. A member of the research team will contact the women who were interested in taking part to discuss the study and assess self-reported eligibility to participate according to the full inclusion and exclusion criteria as detailed below. Eligible individuals who wish to participate will be booked in for a face-to-face baseline appointment. The aim is to recruit women as early in pregnancy as possible, therefore we will aim to book the baseline appointment for the trial within two weeks after the dating scan. However, recognise this extra appointment at short notice may be challenging, and during this feasibility trial we will allow women to attend their baseline appointment up to 20 weeks' gestation. This allows at least a month to practise the dietary changes and assess whether the progression criteria are met. In addition, we will monitor the timing of the baseline appointment to check whether setting a limit at 14 weeks' gestation for recruitment to any future definitive trial, would be feasible. For those who decline to participate, permission will be sought to record the barriers to participation.

Eligibility criteria

Inclusion criteria

- Pregnant women <20 weeks' gestation.
- Aged ≥18 years.
- BMI≥30kg/m^2 measured at booking appointment.
- Planned antenatal care and delivery at the study setting.

For peer review only - http://bmjopen.bmj.com/site/about/guidelines.xhtml

Exclusion criteria

The participant may be excluded if any of the following apply:

- Severe congenital fetal anomaly (confirmed by ultrasound).
- Planned termination of pregnancy or ectopic pregnancy.
- Pre-pregnancy diagnosis of diabetes or impaired glucose tolerance or taking metformin.
- Renal disease.
- Severe liver disease.
- Organ transplant.
- Cardiac failure (Grade II New York Heart Association, and more severe).[35]
- Severe neurological disorder (including epilepsy).
- Severe psychiatric disease requiring in-patient admission.
- GDM diagnosis at the baseline study visit according to the National Institute for Health and Care Excellence (NICE) diagnostic criteria: a fasting plasma glucose level of 5.6mmol/L or above, or a 2-hour plasma glucose level of 7.8mmol/L and above.[36]
- History or presence of eating disorder.
- Hyperemesis gravidarum.
- Unable to understand spoken and written English.
- Previous bariatric surgery.
- Women participating in other intervention research studies.
- Any other significant disease or disorder which may either put the participants at risk because of participation in the trial, or may influence the result of the study, or the participant's ability to participate in the study.

For peer review only

For peer review only - http://bmjopen.bmj.com/site/about/guidelines.xhtml

Study procedures

The baseline visit will take place before 20 weeks' gestation, at the hospital, with a member of the research team, and lasts for approximately 2 hours. Written informed consent will be sought and eligibility for inclusion will be formally assessed. All participants will complete the following baseline assessments: blood pressure, weight and height measurements, a two-hour 75 gram Oral Glucose Tolerance Test (OGTT), a quality of life questionnaire, and measurement of their carbohydrate intake as part of a broader assessment of their dietary intake via an online questionnaire. The participants will be randomised to one of the two trial arms. Participants in the intervention group will proceed to receive the intervention session. Due to the nature of the OGTT test and exclusion of women with GDM confirmed on this baseline test, full eligibility can only be confirmed after the visit, therefore after randomisation. Whilst there is controversy over whether true GDM can be diagnosed prior to 24 weeks' gestation with existing criteria,[37] the clinical care team advised that women meeting the NICE criteria for GDM at the baseline visit,[36] should be considered to have GDM and be treated as such. All confirmed eligible participants will be invited to attend a further 2-hour research visit at 24-28 weeks' gestation where the same research measurements will be collected as a follow-up, and a second OGTT will be completed. Maternal and neonatal outcomes will be extracted from medical records after delivery. The study flow is in **Figure 1**.

Sample size

This feasibility study is not powered to detect a statistically significant difference in clinical effectiveness between the trial arms. Instead, we will test for reductions in carbohydrate intake as preliminary evidence of the adoption of the reduced carbohydrate diet to an extent that is expected to alter health outcomes. The average total carbohydrate intake in women who are not pregnant is about 230g/day.[34] We have set the sample size to be able to detect a reduced target intake at 130-

For peer review only - http://bmjopen.bmj.com/site/about/guidelines.xhtml

150g, i.e. ~`140g of total carbohydrate/day at follow-up, with 95% Confidence Interval (CI) excluding 170g/day. Progression criteria to a full trial also include adequate retention of participants as a measure of feasibility. We consider that a minimum of 80% of participants need to complete an OGTT at 24-28 weeks' gestation, similar to previous trials, with 95% CI excluding 70%.[12], [16] Both progression criteria thresholds are satisfied with a sample size of 60 participants allocated in 2:1 ratio, intervention: control. Unequal randomisation allows us to have sufficient participants in the intervention arm for the qualitative study.

Randomisation

Eligible participants will be randomised to one of the two treatment groups using permuted block randomisation with random block sizes of 3 and 6. An independent researcher will generate the allocation sequence and assignment will be revealed once eligibility has been confirmed using an online software (Study Randomizer) to ensure allocation concealment.[38] Due to the nature of this study, it will not be possible to blind the participants, clinicians or the study team to the treatment allocation beyond this point.

Intervention and control

Intervention: the RECORD programme

The intervention comprises a moderately reduced-carbohydrate behavioural intervention, combining dietary advice, with structured written dietary information. It aims to improve participants' adherence to the programme through a simple behavioural support programme, which includes goal-setting, planning, feedback and problem-solving. The intervention will be delivered during a 30-minute session with a healthcare professional (HCP) during the baseline visit, where the intervention materials and advice will be provided, and will be supplemented with up to 6 brief telephone support sessions of up to 15 minutes depending on patient need. Participants in this group will be advised to follow the intervention until delivery.

For peer review only - http://bmjopen.bmj.com/site/about/guidelines.xhtml

This intervention was developed based on a review of the literature, study protocols, qualitative data of similar interventions, and interviews with a diabetes-specialist dietitian, two obstetricians, a research midwife, and a patient representative with previous GDM. A group of women affected by GDM, also helped to shape the ideas for this research, and to determine the questions we are trying to answer, and the type of dietary information and support they felt would be helpful.

The dietary component of this intervention consists of a moderately reduced-carbohydrate diet (130-150g of total carbohydrate per day), with no additional energy restriction. This is in line with the dietary advice provided in previous GDM trials, as well as for women diagnosed with GDM at the hospital. The difference here, is that the reduced-carbohydrate advice is given earlier in pregnancy, to assess whether it has benefits for women at increased risk of GDM. The core principles include:

a) **REFRAIN** from having sugary foods and drinks (e.g. biscuits, confectionary, juice, sugary fizzy drinks), with the exception of dairy and limited fruit intake;

b) **REDUCE** the amount of starchy carbohydrates to a moderate level, by having smaller portions of foods such as rice, pasta, bread, grains, potato, yams, cassava, plantain, at main meals;

c) **REPLACE** refined starchy carbohydrates with wholegrain, unrefined versions, and sugary foods and drinks, with lower-carbohydrate, and lower, or no-sugar alternatives.

We anticipate that women in this study will consume more than the target 130-150g of carbohydrate per day at baseline, but if the diet questionnaire reveals that a participant has carbohydrate intake already close to the recommended level, the advice given will ensure that their intake is maintained through healthy food choices in line with the RECORD diet principles. Standard healthy eating advice during pregnancy is also included, e.g. high intake of vegetables, moderate intake of lean protein, and guidance on foods that should be avoided during pregnancy. Information

For peer review only

For peer review only - http://bmjopen.bmj.com/site/about/guidelines.xhtml

is discussed regarding the importance of controlling GWG, and women in this group are advised to weigh themselves once a week.

The development of this intervention was also informed by behavioural frameworks aiming to promote successful behaviour change. These are: the Capability, Opportunity, and Motivation-Behavioural model (COM-B), the Theoretical Domains Framework (TDF), and the Behaviour Change Wheel (BCW).[39], [40] By utilising these frameworks, we targeted the components of psychological capability (e.g. knowledge/understanding, skills, decision making, self-regulation), physical and social opportunity (e.g. social influences and support, and environmental changes), and reflective motivation (e.g. knowledge/understanding, confidence regarding capabilities, beliefs regarding risks, intentions, plans). To address these components, the intervention includes:

- A consultation including the essential principles of the diet as described above, as well as the rationale behind the intervention and how it may work in controlling blood glucose levels, and attenuating GWG.
- Written resources to enhance participants' confidence and skills in following the programme, and to facilitate decision making, including advice on food selection, meal and snack suggestions, and suggested recipes.
- Setting personalised realistic goals, by letting participant choose what changes they are comfortable making and the steps to achieve them. Participants can choose to use a "Personal action plan" booklet to set their goals and reflect on their progress, as well as goal progression checklists, and self-weighing charts, however, these are also addressed during the support sessions.
- Structured healthcare professional follow-up to provide support, contingency planning, problem-solving, planning of social support, advice on lapses, and help with environmental or emotional triggers, and to provide feedback on progress. Additionally, participants are

For peer review only - http://bmjopen.bmj.com/site/about/guidelines.xhtml

able to self-monitor their health via home finger-prick testing of their blood glucose and ketones, and by weighing themselves, which provides additional monitoring.

- Provision of feedback on progress and changes in clinical measures as a result of following the intervention, e.g. GWG, capillary blood glucose and ketones, glucose curve from OGTTs, blood pressure.

Control

All women will receive routine antenatal care, which usually includes one-off face-to-face routine care NHS dietary advice during their booking appointment with a midwife. Anecdotal reports suggest this is focused on foods to be avoided during pregnancy for safety of the fetus, rather than healthy eating or weight control advice. This will have been given prior to randomisation in the study. Participants allocated to the control group will receive no additional advice beyond this.

Patient and public involvement

We convened two focus groups with eight women recruited from the local antenatal clinic, national GDM support website, and a social media forum. The first group helped to prioritise and refine the research question. In a second phase, women were provided with an outline of the study proposal, and were asked to provide further comments. From both phases, it was clear that women felt upset, shocked and anxious following a diagnosis of GDM, and highlighted a need for preventative interventions that could be implemented earlier in pregnancy. Participants also highlighted the importance of including neonatal outcomes in addition to the maternal outcomes. Women were interested in taking part in clinical trials aiming to prevent, treat or manage GDM, however, felt that that attending extra appointments for study visits and in particular having to use the parking at the hospital, would be a major barrier to participation. As a final stage, a patient representative provided feedback on the intervention materials to help make them clearer and more concise and continues to support the study team.

For peer review only

For peer review only - http://bmjopen.bmj.com/site/about/guidelines.xhtml

Outcomes

Primary

The primary objective of this study is to test whether this reduced carbohydrate programme is feasible and leads to a reduction in carbohydrate intake in women who are pregnant and at risk of developing GDM. This feasibility measures will further determine whether to progress to a full RCT.

Progression criteria will be based on a traffic light system to decide whether to proceed to a full RCT (green), proceed with amendments for remediable issues (amber), or to not to proceed (red). (**Figure 2**).[41] The progression criteria will be:

1. **Adoption of the intervention:** that the intervention group reduces their total carbohydrate consumption by the time of follow-up, with green light for 95% CI of average intake that does not cross 170g/day. (Evaluated as part of a broader assessment of dietary intake through an online 24-hour dietary recall questionnaire at the baseline and follow-up visits – detailed below).
2. **Retention rate:** proportion of all participants who complete an OGTT at the follow-up time point, with green light for 95% CI of average completion rate that does not cross 70%. (Evaluated by documentation at 24-28 weeks' gestation; assessed as the proportion of participants for whom OGTT measurements are available).

Secondary

- Change in fasting plasma glucose, 1-hour and 2-hour glucose. (OGTT at baseline and follow-up visit)
- Change in HbA1c, fasting plasma insulin and insulin resistance (HOMA-IR). (Blood samples collected as part of OGTT at baseline and follow-up visit)

For peer review only - http://bmjopen.bmj.com/site/about/guidelines.xhtml

- Proportion of participants who are diagnosed with GDM. (Documentation at baseline and follow-up visit)
- GWG: weight change from baseline until before delivery. If pre-delivery weight is not available, we will use weight at 36 weeks' gestation collected as part of routine practice.
- Proportion of participants meeting the Institute of Medicine recommendations for GWG.[42]
- Dietary intake: total carbohydrate intake (g/day) and total energy intake (MJ/day), percentage of daily total energy intake from carbohydrates. (Dietary composition analysis at baseline and follow-up visit– see details below)
- Distribution of fasting capillary blood glucose and ketone levels across gestational age from baseline until before delivery.

Process

- Number of eligible women who are approached and enrolled into the study per month. (Documentation)
- Percentage of intervention participants who complete the initial and support sessions. (Documentation at each relevant time point)
- Percentage of participants in each group who attend the follow-up visit. (Documentation)
- Percentage of participants for whom weight at 36 weeks' gestation is available (usually measured as part of a routine hospital appointment).
- Proportion of participants who record their blood glucose and blood ketones every week. (Documentation from baseline until before delivery)
- Proportion of participants in the intervention group who self-monitor their weight every week. (Documentation from baseline until before delivery)
- Proportion of participants in the control group who, after randomisation, follow a reduced-carbohydrate diet (contamination of the control group). (Dietary composition analysis at baseline and follow-up visit– see details below)

For peer review only

For peer review only - http://bmjopen.bmj.com/site/about/guidelines.xhtml

- Proportion of essential elements included in intervention delivery sessions (main and support sessions). (Evaluation of audio-recorded sessions against a checklist)

Exploratory outcomes:

- Further dietary changes: fibre intake, percentage of daily total energy intake from total fat, saturated fat and protein. (Dietary composition analysis at baseline and follow-up visit – see details below)
- Changes in systolic and diastolic blood pressure from baseline to follow-up.
- Changes in pregnancy-related outcomes e.g. gestational hypertension and preeclampsia, requirement for insulin or oral diabetic medication, mode of delivery, induction of labour, gestational age at delivery, birthweight, sex of baby, rates of macrosomia (>4500g),[43] large-for-gestational age (>90th percentile) and small-for-gestational age infant (<10th percentile),[43] neonatal adverse events, e.g. neonatal intensive care unit admission, shoulder dystocia/birth trauma, neonatal hypoglycaemia requiring intravenous glucose, neonatal hyper bilirubinaemia requiring phototherapy. (Extraction from medical record following delivery)
- Change in quality of life from baseline to follow-up.
- Participant acceptability of the intervention and the materials, overall experience of the intervention, barriers and facilitators of adherence (qualitative interviews).

Measurements

A schedule of measurements can be found in **Table 1**.

Sociodemographic characteristics

Participants will be asked to self-report highest education level, employment status and ethnicity. Age will be accessed from the medical records.

For peer review only

For peer review only - http://bmjopen.bmj.com/site/about/guidelines.xhtml

Physical measurements

Height will be measured using stadiometers, to the nearest 0.1 cm. Weight will be measured to the nearest 0.1kg using a digital scale (Active Era, BS-03S) at the study visits. For self-weighing at home, participants in the intervention group will be instructed to use their own digital scales or will be given a set by the research team if they do not own a set. They can either text the measurements to the research team or log them manually onto a mobile application (detailed below). At 36-weeks' gestation, women should be weighed during a routine antenatal appointment at their primary care practice, and digital scales are used for this. If pre-delivery weight is available, digital scales are also used for this. Blood pressure will be measured in duplicate using a digital blood pressure machine (Omron, M3) after 5 min seated rest, with at least 1 min between each measurements.

OGTT and remote glucose and ketone monitoring

Women are asked to attend the study visits after an overnight fast, and to refrain from smoking, vaping, using nicotine replacement therapy or chewed sugary gum for at least 8 hours before. A fasting venous blood sample will be collected for the determination of glucose, HbA1c, insulin concentrations. Participants are provided with a 75g glucose load (Polycal, Nutricia). Further venous blood samples will be collected at 1 hour and 2 hours post-drink, for the determination of glucose concentrations.

All participants are provided with a Bluetooth-enabled capillary blood glucose and ketone monitoring kit (4SURE Smart Duo, Nipro Diagnostics Ltd). The researcher provides a short demonstration and information flyer on how to collect and record the measurements, and advises participants to take a fasting capillary glucose and ketone measurement twice a week. The monitor is connected remotely to a mobile application (Diabetes: M, Sirma Medical Systems), allowing the research team to monitor the readings remotely and escalate concerns to the clinical team. We did not provide cut-offs to participants for fasting capillary glucose and ketone levels, due to lack of level

For peer review only

For peer review only - http://bmjopen.bmj.com/site/about/guidelines.xhtml

references specific to pregnancy and before GDM diagnosis. Two obstetricians from the study team who are also members of the clinical care team interpreted the measurements on an individual basis and/or based on evidence from non-pregnant populations. The collection of this data during the feasibility study will guide thresholds that can be set for any future trial.

Carbohydrate consumption

Participants will complete at baseline (<20 weeks' gestation) and follow-up (24-28 weeks' gestation) an online multiple-pass 24-hour recall, validated against interviewer-led multiple-pass recalls in adolescents and adults (Intake 24, Newcastle University).[44], [45] This will be used to primarily assess carbohydrate consumption, but also broader dietary intake. By the time participants enrol in the study (mid-pregnancy), their dietary intake is likely to have stabilised compared to the fluctuations usually observed in the first trimester due to pregnancy symptoms. Intake 24 asks participants to report whether a reporting day was typical with regards to their dietary intake, and this will be taken into account in the analysis.

Quality of life

Participants will be asked to fill in a questionnaire about their quality of life, via the European Quality of Life Five Dimension questionnaire and subscale.[46]

Retention and withdrawal

This study has been designed to run alongside routine antenatal appointments with remote support elements, to minimise the burden associated with attending extra visits. The brief telephone support sessions with a dietitian will act as prompts for the participants to engage with the intervention and self-monitoring. Participants are asked to attend one visit in addition to routine antenatal care. All of the women who take part would be offered an OGTT at 24-28 weeks of pregnancy for the diagnosis of GDM. The 24-28-week follow-up study visit and OGTT, replaces the

For peer review only - http://bmjopen.bmj.com/site/about/guidelines.xhtml

routine care OGTT, and the results are shared with the clinical care team. To compensate for the time and inconvenience in taking part, we offer participants financial incentives for their participation.

Each participant will have the right to withdraw from the study at any time, or may be withdrawn at the discretion of the research or clinical care team. Participants will have the right to withdraw their collected data if they wish. Withdrawn eligible and enrolled participants will not be replaced in this feasibility study. We will only replace participants whose baseline OGTT is abnormal, as they will have consented and been randomised before OGTT results become available, but they will have essentially failed the full screening.

GDM diagnosis

Participants who develop GDM or who are diagnosed with GDM at the 24-28 weeks' follow-up visit, will be referred to the clinical care team and offered the current standard medical treatment. In line with the NICE guidelines, this includes monitoring of blood glucose (two of the fasting measurements will continue to be used in the study), and diet and physical activity advice for the management of GDM.[36] More specifically, women with GDM are advised to manage carbohydrate intake, to limit their weight gain until delivery, and are encouraged to do 150 minutes of moderate intensity activity per week. Women in the intervention group, can choose to continue following the RECORD programme until delivery (at the discretion of the clinical care team). Women in the control group will receive advice to reduce their carbohydrate intake at this stage, and this will be noted. All participants with GDM, will be encouraged to continue monitoring their ketone levels twice a week for the remaining of pregnancy.

For peer review only - http://bmjopen.bmj.com/site/about/guidelines.xhtml

Statistical analysis

Descriptive statistics will be used to report the baseline characteristics of each trial arm. Continuous variables will be reported using mean/median and standard deviation/range, while categorical variables will be reported using frequencies and percentages.

Since this is a feasibility study, the primary outcomes are the main progression criteria which will inform the design of a future RCT, powered to detect a significant difference in the efficacy of the intervention in preventing GDM. Descriptive statistics with 95% CIs will be used to analyse and report the progression criteria. For the analysis of the secondary, process-related and exploratory measures, all data collected up to each time point will be used. Descriptive comparative statistics will be used to analyse and report these measures along with 95% CIs (e.g. difference in means or proportions between baseline and follow-up for each group or between the two groups). Data from audio-recorded and transcribed qualitative interviews on acceptability and experience of the intervention by participants, will be analysed using thematic analysis and reported descriptively.

Qualitative study

A qualitative sub-study with participants randomised to the RECORD group will explore participants' acceptability of the intervention and their overall experience of the study, explore barriers and facilitators to adherence, and identify suggestions that could refine the intervention should it progress to a full RCT.

During the follow-up study visit a member of the research team will provide participants allocated to the intervention group, with a separate participant information sheet about the qualitative interviews. If participants are happy to have this in-depth discussion about their experiences of the RECORD intervention, a member of the research team will arrange a convenient day and time for a telephone interview. The participants will consent verbally on the phone and the researcher will fill in written consent form. The interviews will last approximately 30 minutes and all

For peer review only

For peer review only - http://bmjopen.bmj.com/site/about/guidelines.xhtml

interviews will be audio-recorded and transcribed verbatim. Data will be analysed using thematic analysis. We will conduct interviews with up to 30 participants, or until data saturation is reached.

Trial management and monitoring

The day-to-day management of the study will be coordinated by MM. As this is a small feasibility study, with no adverse event monitoring or stopping rules, there are no monitoring committees in place. A trial management group consisting of the authors of this paper, a research midwife, and a patient representative, will have oversight of the trial and meet bi-monthly to discuss progress and decide on actions to be taken.

Adverse events

We will record and report all serious adverse events (SAEs) according to Good Clinical Practice (GCP), and Health Research Authority's (HRA) processes. Recording of SAEs will start immediately following enrolment into the study and will be continued until the end of the study.

Data management

Data will be kept in accordance with GCP, the Data Protection Act 2018, and the General Data Protection Regulation. Two separate databases will be created, one containing all participant identifiable information, and one where data collected during the research procedures will be entered in an anonymised manner, using a unique participant ID (electronic CRFs). Anonymised data from the online diet questionnaire will be downloaded from the Intake24 website and added to the second database. Quality of life anonymised data will be collected on hard copy questionnaires and then entered into the second database. The two databases, as well as anonymised recordings and transcriptions from the main and support intervention sessions, will be password-protected and stored on the secure drive of Nuffield Department of Primary Care Health Sciences, and will only be accessible to authorised members of the research team. After publication of the results has been

For peer review only - http://bmjopen.bmj.com/site/about/guidelines.xhtml

sent out to participants, their contact details will be deleted/destroyed. We will retain the anonymised research data for future secondary analyses.

Direct access to study data will be granted to authorised representatives from the Sponsor and host institution for monitoring and/or audit of the study to ensure compliance with regulations. Otherwise, confidentiality will be maintained and no-one outside the research team will have access to the database.

Ethics and dissemination

This protocol (Version 1.3, 12 July 2021) was reviewed and approved by the South-Central Oxford B Research Ethics Committee NHS National Research Ethics Committee and the Health Research Authority (Reference: 20/SC/0442), and prospectively registered on ISRCTN (ISRCTN16235884). Any substantial changes to the protocol will be notified and reviewed by NHS Research Ethics Committee, the Health Research Authority and the Sponsor (University of Oxford), and trial registry will be updated accordingly. The findings of this feasibility study will be submitted for publication in a peer-reviewed journal, and presented at conferences, to disseminate the results to academic and health professional audiences, and made available to participants and to the wider public on our website at the time of publication.

For peer review only

Sponsor: This trial is sponsored by the University of Oxford, Clinical Trials and Research Governance, Joint Research Office, Block 60, Churchill Hospital, Old Road, Headington, Oxford, OX3 7LE, UK.

Funding: This trial is funded by the NIHR Oxford Biomedical Research Centre (IS-BRC-1215-20008). MM's time on this project is funded by Oxford-Medical Research Council Doctoral Training Partnership (MR/N013468/1). JEH is supported by a UK Research and Innovation Future Leaders Fellowship (MR/T040750/1).

Disclaimer: The views expressed are those of the authors and not necessarily those of the NHS, the NIHR, the University of Oxford or the Department of Health. The sponsor or funding bodies had no role in the study design, collection, management, analysis, and interpretation of data; writing of the report; and the decision to submit the report for publication.

Contributions: MM, NA and SAJ developed the concept for the study and wrote the first draft of the protocol with input from LM and JEH on clinical aspects. MM, NA and SAJ prepared the study documents and coordinated the HRA and ethics application. The sponsor has reviewed all participant-facing documents as part of the ethics application. LM, JEH, and PD approved the final protocol. MM, NA, SAJ, and PD were involved in the detailed design of the intervention. MM and PD developed the intervention materials with input from NA, SAJ, LM, JEH and AW. MM drafted the manuscript for publication, with input from NA and SAJ. LM, JEH, PD, and AW reviewed, provided input and approved the final manuscript.

Competing interests:

LM is a part-time employee of EMIS Group plc'

Acknowledgements:

We thank Sr Yvonne Kenworthy, Research Midwife, for providing feedback on the intervention materials.

For peer review only

Figure 1. Study flow

Legend: BMI: body mass index; OGTT: oral glucose tolerance test; GDM: gestational diabetes

Figure 2. Progression criteria

Legend: OGTT: Oral Glucose Tolerance Test

For peer review only

For peer review only - http://bmjopen.bmj.com/site/about/guidelines.xhtml

Table 1. Schedule of study measurements

	STUDY PERIOD								
	Enrolment	Allocation	Study visits/sessions						Delivery
**TIMEPOINT**	Baseline = Week 0 (<20 weeks' gestation)	Week 0	Week 2	Week 4	~Week 8	Follow-up visit = ~Week 12 (24-28 weeks' gestation)	~Week 16	~Week 20	Week 24
**ENROLMENT:**									
**Eligibility screening**	X								
**Informed consent**	X								
**Randomisation**	X								
**TRIAL ARMS:**									
**RECORD programme**	X	X				X			X
**+ telephone support**			X	X	X	X	X	X	
**Usual care**	X	X				X			X
**ASSESSMENTS:**									
**Demographics**	X								
**Weight**	X					X			
**Height**	X								
**Blood pressure**	X					X			
**OGTT**	X					X			
**EQ-5D**	X					X			
**Self-completed 24-hour dietary recall**	X					X			
**Fasting plasma insulin**	X					X			
**HbA1c**	X					X			
**Self-monitoring of fasting capillary glucose**		Twice a week for the study duration							
**Self-monitoring of fasting capillary ketones**		Twice a week for the study duration							
**Self-weighing (intervention group)**		Once a week for the study duration							
**SAEs**									
**Maternal and neonatal outcomes**									X

EQ-5D: EuroQol – 5 Dimensions questionnaire; OGTT: Oral Glucose Tolerance Test; SAEs: Serious Adverse Events

For peer review only - http://bmjopen.bmj.com/site/about/guidelines.xhtml

For peer review only

References

1. American Diabetes Association. 2. Classification and Diagnosis of Diabetes: Standards of Medical Care in Diabetes-2020. *Diabetes Care* 2020;43(Suppl 1):S14-S31. doi: 10.2337/dc20-S002 [published Online First: 2019/12/22]
2. The HAPO Study Cooperative Research Group, Metzger BE, Lowe LP, et al. Hyperglycemia and adverse pregnancy outcomes. *N Engl J Med* 2008;358(19):1991-2002. doi: 10.1056/NEJMoa0707943 [published Online First: 2008/05/09]
3. Vounzoulaki E, Khunti K, Abner SC, et al. Progression to type 2 diabetes in women with a known history of gestational diabetes: systematic review and meta-analysis. *BMJ* 2020;369:m1361. doi: 10.1136/bmj.m1361 [published Online First: 2020/05/15]
4. Daly B, Toulis KA, Thomas N, et al. Increased risk of ischemic heart disease, hypertension, and type 2 diabetes in women with previous gestational diabetes mellitus, a target group in general practice for preventive interventions: A population-based cohort study. *PLoS Med* 2018;15(1):e1002488. doi: 10.1371/journal.pmed.1002488 [published Online First: 2018/01/18]
5. Lowe WL, Jr., Lowe LP, Kuang A, et al. Maternal glucose levels during pregnancy and childhood adiposity in the Hyperglycemia and Adverse Pregnancy Outcome Follow-up Study. *Diabetologia* 2019;62(4):598-610. doi: 10.1007/s00125-018-4809-6 [published Online First: 2019/01/17]
6. Kramer CK, Campbell S, Retnakaran R. Gestational diabetes and the risk of cardiovascular disease in women: a systematic review and meta-analysis. *Diabetologia* 2019;62(6):905-14. doi: 10.1007/s00125-019-4840-2 [published Online First: 2019/03/08]
7. Boney CM, Verma A, Tucker R, et al. Metabolic syndrome in childhood: association with birth weight, maternal obesity, and gestational diabetes mellitus. *Pediatrics* 2005;115(3):e290-6. doi: 10.1542/peds.2004-1808 [published Online First: 2005/03/03]
8. Yu Y, Arah OA, Liew Z, et al. Maternal diabetes during pregnancy and early onset of cardiovascular disease in offspring: population based cohort study with 40 years of follow-up. *BMJ* 2019;367:l6398. doi: 10.1136/bmj.l6398 [published Online First: 2019/12/06]
9. Lowe WL, Jr., Scholtens DM, Kuang A, et al. Hyperglycemia and Adverse Pregnancy Outcome Follow-up Study (HAPO FUS): Maternal Gestational Diabetes Mellitus and Childhood Glucose Metabolism. *Diabetes Care* 2019;42(3):372-80. doi: 10.2337/dc18-1646 [published Online First: 2019/01/19]
10. Farrar D, Simmonds M, Griffin S, et al. The identification and treatment of women with hyperglycaemia in pregnancy: an analysis of individual participant data, systematic reviews, meta-analyses and an economic evaluation. *Health Technol Assess* 2016;20(86):1-348. doi: 10.3310/hta20860 [published Online First: 2016/12/06]
11. Brunner S, Stecher L, Ziebarth S, et al. Excessive gestational weight gain prior to glucose screening and the risk of gestational diabetes: a meta-analysis. *Diabetologia* 2015;58(10):2229-37. doi: 10.1007/s00125-015-3686-5 [published Online First: 2015/07/05]
12. Poston L, Bell R, Croker H, et al. Effect of a behavioural intervention in obese pregnant women (the UPBEAT study): a multicentre, randomised controlled trial. *Lancet Diabetes Endocrinol* 2015;3(10):767-77. doi: 10.1016/S2213-8587(15)00227-2 [published Online First: 2015/07/15]
13. Dodd JM, Cramp C, Sui Z, et al. The effects of antenatal dietary and lifestyle advice for women who are overweight or obese on maternal diet and physical activity: the LIMIT randomised trial. *BMC Med* 2014;12:161. doi: 10.1186/s12916-014-0161-y [published Online First: 2014/10/16]
14. Koivusalo SB, Rönö K, Klemetti MM, et al. Gestational Diabetes Mellitus Can Be Prevented by Lifestyle Intervention: The Finnish Gestational Diabetes Prevention Study (RADIEL): A

For peer review only

Randomized Controlled Trial. *Diabetes Care* 2016;39(1):24-30. doi: 10.2337/dc15-0511 [published Online First: 2015/08/01]
15. Simmons D, Jelsma JG, Galjaard S, et al. Results From a European Multicenter Randomized Trial of Physical Activity and/or Healthy Eating to Reduce the Risk of Gestational Diabetes Mellitus: The DALI Lifestyle Pilot. *Diabetes Care* 2015;38(9):1650-6. doi: 10.2337/dc15-0360 [published Online First: 2015/06/27]
16. Al Wattar BH, Dodds J, Placzek A, et al. Mediterranean-style diet in pregnant women with metabolic risk factors (ESTEEM): A pragmatic multicentre randomised trial. *PLoS Med* 2019;16(7):e1002857. doi: 10.1371/journal.pmed.1002857 [published Online First: 2019/07/25]
17. Luoto R, Kinnunen TI, Aittasalo M, et al. Primary prevention of gestational diabetes mellitus and large-for-gestational-age newborns by lifestyle counseling: a cluster-randomized controlled trial. *PLoS Med* 2011;8(5):e1001036. doi: 10.1371/journal.pmed.1001036 [published Online First: 2011/05/26]
18. Bruno R, Petrella E, Bertarini V, et al. Adherence to a lifestyle programme in overweight/obese pregnant women and effect on gestational diabetes mellitus: a randomized controlled trial. *Matern Child Nutr* 2017;13(3) doi: 10.1111/mcn.12333 [published Online First: 2016/09/21]
19. Harrison CL, Lombard CB, Strauss BJ, et al. Optimizing healthy gestational weight gain in women at high risk of gestational diabetes: a randomized controlled trial. *Obesity (Silver Spring)* 2013;21(5):904-9. doi: 10.1002/oby.20163 [published Online First: 2013/06/21]
20. Markovic TP, Muirhead R, Overs S, et al. Randomized Controlled Trial Investigating the Effects of a Low-Glycemic Index Diet on Pregnancy Outcomes in Women at High Risk of Gestational Diabetes Mellitus: The GI Baby 3 Study. *Diabetes Care* 2016;39(1):31-8. doi: 10.2337/dc15-0572 [published Online First: 2015/07/18]
21. Hui A, Back L, Ludwig S, et al. Lifestyle intervention on diet and exercise reduced excessive gestational weight gain in pregnant women under a randomised controlled trial. *BJOG* 2012;119(1):70-7. doi: 10.1111/j.1471-0528.2011.03184.x [published Online First: 2011/10/25]
22. Petrella E, Malavolti M, Bertarini V, et al. Gestational weight gain in overweight and obese women enrolled in a healthy lifestyle and eating habits program. *J Matern Fetal Neonatal Med* 2014;27(13):1348-52. doi: 10.3109/14767058.2013.858318 [published Online First: 2013/11/02]
23. Assaf-Balut C, Garcia de la Torre N, Duran A, et al. A Mediterranean diet with additional extra virgin olive oil and pistachios reduces the incidence of gestational diabetes mellitus (GDM): A randomized controlled trial: The St. Carlos GDM prevention study. *PLoS One* 2017;12(10):e0185873. doi: 10.1371/journal.pone.0185873 [published Online First: 2017/10/20]
24. Wolff S, Legarth J, Vangsgaard K, et al. A randomized trial of the effects of dietary counseling on gestational weight gain and glucose metabolism in obese pregnant women. *Int J Obes (Lond)* 2008;32(3):495-501. doi: 10.1038/sj.ijo.0803710 [published Online First: 2008/01/30]
25. Griffith RJ, Alsweiler J, Moore AE, et al. Interventions to prevent women from developing gestational diabetes mellitus: an overview of Cochrane Reviews. *Cochrane Database Syst Rev* 2020;6:CD012394. doi: 10.1002/14651858.CD012394.pub3 [published Online First: 2020/06/12]
26. International Weight Management in Pregnancy Collaborative Group. Effect of diet and physical activity based interventions in pregnancy on gestational weight gain and pregnancy outcomes: meta-analysis of individual participant data from randomised trials. *BMJ* 2017;358:j3119. doi: 10.1136/bmj.j3119 [published Online First: 2017/07/21]
27. Bush H WR, Bradby H, Anderson A, Lean M. . Family hospitality and ethnic tradition among South Asian, Italian and general population women in the West of Scotland. *Social Health Ill* 1998;20(3):351-80.

For peer review only

For peer review only - http://bmjopen.bmj.com/site/about/guidelines.xhtml

28. Drewnowski A, Darmon N. Food choices and diet costs: an economic analysis. *J Nutr* 2005;135(4):900-4. doi: 10.1093/jn/135.4.900 [published Online First: 2005/03/30]
29. Moore SE, McEvoy CT, Prior L, et al. Barriers to adopting a Mediterranean diet in Northern European adults at high risk of developing cardiovascular disease. *J Hum Nutr Diet* 2018;31(4):451-62. doi: 10.1111/jhn.12523 [published Online First: 2017/11/22]
30. Singh M, Hung ES, Cullum A, et al. Lower carbohydrate diets for adults with type 2 diabetes. *Br J Nutr* 2021:1-6. doi: 10.1017/s0007114521002373 [published Online First: 2021/11/02]
31. Feinman RD, Pogozelski WK, Astrup A, et al. Dietary carbohydrate restriction as the first approach in diabetes management: critical review and evidence base. *Nutrition* 2015;31(1):1-13. doi: 10.1016/j.nut.2014.06.011 [published Online First: 2014/10/08]
32. Frise CJ, Mackillop L, Joash K, et al. Starvation ketoacidosis in pregnancy. *Eur J Obstet Gynecol Reprod Biol* 2013;167(1):1-7. doi: 10.1016/j.ejogrb.2012.10.005 [published Online First: 2012/11/08]
33. Simmons D, Devlieger R, van Assche A, et al. Effect of Physical Activity and/or Healthy Eating on GDM Risk: The DALI Lifestyle Study. *J Clin Endocrinol Metab* 2017;102(3):903-13. doi: 10.1210/jc.2016-3455 [published Online First: 2016/12/10]
34. Roberts CS, Maplethorpe, N, Cox N, et al. National Diet and Nutrition Survey: Results from Years 7 and 8 (combined) of the Rolling Programme (2014/2015 to 2015/2016). London: Public Health England; 2018.
35. New York Heart Association. Inc. Diseases of the Heart and Blood Vessels. Nomenclature and Criteria for diagnosis. 6th ed. Boston1964:114.
36. NICE. Gestational diabetes: risk assessment, testing, diagnosis and management. Available from: https://pathways.nice.org.uk/pathways/diabetes-in-pregnancy/gestational-diabetes-risk-assessment-testing-diagnosis-and-management#content=view-node%3Anodes-risk-assessment. Accessed August 2021.
37. US Preventative Services Task Force. Screening for Gestational Diabetes: US Preventive Services Task Force Recommendation Statement. *JAMA* 2021;326(6):531-38. doi: 10.1001/jama.2021.11922
38. Randomizer S. Available from: https://www.studyrandomizer.com/. Accessed May 2021.
39. Michie S, van Stralen MM, West R. The behaviour change wheel: a new method for characterising and designing behaviour change interventions. *Implement Sci* 2011;6:42. doi: 10.1186/1748-5908-6-42 [published Online First: 2011/04/26]
40. Michie S, Johnston M, Abraham C, et al. Making psychological theory useful for implementing evidence based practice: a consensus approach. *Qual Saf Health Care* 2005;14(1):26-33. doi: 10.1136/qshc.2004.011155 [published Online First: 2005/02/05]
41. Avery KN, Williamson PR, Gamble C, et al. Informing efficient randomised controlled trials: exploration of challenges in developing progression criteria for internal pilot studies. *BMJ Open* 2017;7(2):e013537. doi: 10.1136/bmjopen-2016-013537 [published Online First: 2017/02/19]
42. Rasmussen KM, Catalano PM, Yaktine AL. New guidelines for weight gain during pregnancy: what obstetrician/gynecologists should know. *Curr Opin Obstet Gynecol* 2009;21(6):521-6. doi: 10.1097/GCO.0b013e328332d24e [published Online First: 2009/10/08]
43. Villar J, Cheikh Ismail L, Victora CG, et al. International standards for newborn weight, length, and head circumference by gestational age and sex: the Newborn Cross-Sectional Study of the INTERGROWTH-21st Project. *Lancet* 2014;384(9946):857-68. doi: 10.1016/S0140-6736(14)60932-6 [published Online First: 2014/09/12]
44. Simpson E, Bradley J, Poliakov I, et al. Iterative Development of an Online Dietary Recall Tool: INTAKE24. *Nutrients* 2017;9(2) doi: 10.3390/nu9020118 [published Online First: 2017/02/18]
45. Foster E DJ, Simpson E, et al. Comparison study: INTAKE24 vs Interviewer led recall. Final report, 2014.
46. EuroQoL. EQ-5D. Available from: https://euroqol.org/. Accessed March 2020.

For peer review only - http://bmjopen.bmj.com/site/about/guidelines.xhtml

For peer review only

For peer review only - http://bmjopen.bmj.com/site/about/guidelines.xhtml

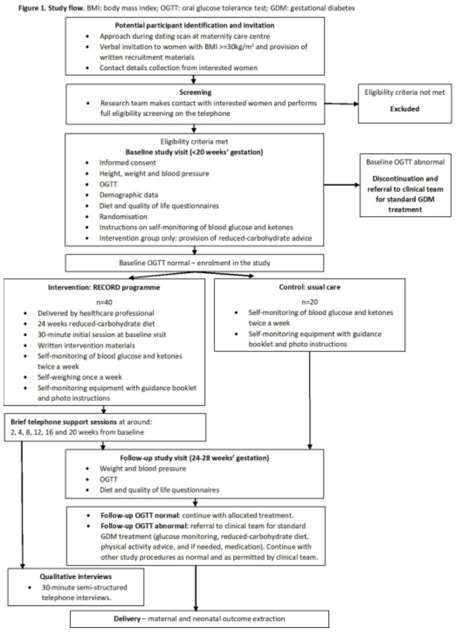

Study flow/BMI: body mass index; OGTT: oral glucose tolerance test; GDM: gestational diabetes

249x155mm (300 x 300 DPI)

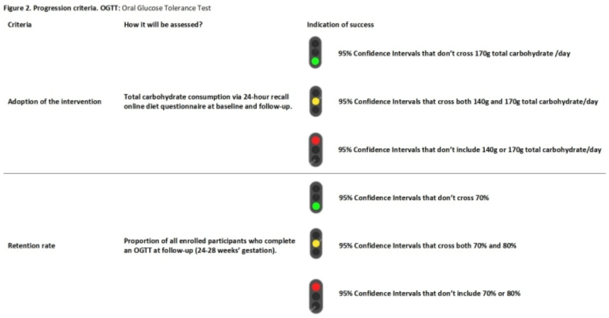

Figure 2. Progression criteria. OGTT: Oral Glucose Tolerance Test

Criteria	How it will be assessed?	Indication of success
Adoption of the intervention	Total carbohydrate consumption via 24-hour recall online diet questionnaire at baseline and follow-up.	95% Confidence Intervals that don't cross 170g total carbohydrate /day<br>95% Confidence Intervals that cross both 140g and 170g total carbohydrate/day<br>95% Confidence Intervals that don't include 140g or 170g total carbohydrate/day
Retention rate	Proportion of all enrolled participants who complete an OGTT at follow-up (24-28 weeks' gestation).	95% Confidence Intervals that don't cross 70%<br>95% Confidence Intervals that cross both 70% and 80%<br>95% Confidence Intervals that don't include 70% or 80%

Progression criteria/OGTT: Oral Glucose Tolerance Test

249x130mm (300 x 300 DPI)

STANDARD PROTOCOL ITEMS: RECOMMENDATIONS FOR INTERVENTIONAL TRIALS

SPIRIT 2013 Checklist: Recommended items to address in a clinical trial protocol and related documents*

Section/item	ItemNo	Description	Lines
**Administrative information**			
Title	1	Descriptive title identifying the study design, population, interventions, and, if applicable, trial acronym	1-3
Trial registration	2a	Trial identifier and registry name. If not yet registered, name of intended registry	43
	2b	All items from the World Health Organization Trial Registration Data Set	43
Protocol version	3	Date and version identifier	486
Funding	4	Sources and types of financial, material, and other support	498-501
Roles and responsibilities	5a	Names, affiliations, and roles of protocol contributors	7-12
	5b	Name and contact information for the trial sponsor	495-496
	5c	Role of study sponsor and funders, if any, in study design; collection, management, analysis, and interpretation of data; writing of the report; and the decision to submit the report for publication, including whether they will have ultimate authority over any of these activities	504-506
	5d	Composition, roles, and responsibilities of the coordinating centre, steering committee, endpoint adjudication committee, data management team, and other individuals or groups overseeing the trial, if applicable (see Item 21a for data monitoring committee)	458-463
**Introduction**			

For peer review only

Background and rationale	6a	Description of research question and justification for undertaking the trial, including summary of relevant studies (published and unpublished) examining benefits and harms for each intervention	58-110
	6b	Explanation for choice of comparators	N/A (usual care)
Objectives	7	Specific objectives or hypotheses	110-119
Trial design	8	Description of trial design including type of trial (eg, parallel group, crossover, factorial, single group), allocation ratio, and framework (eg, superiority, equivalence, noninferiority, exploratory)	120-126 and 200-201
**Methods: Participants, interventions, and outcomes**			
Study setting	9	Description of study settings (eg, community clinic, academic hospital) and list of countries where data will be collected. Reference to where list of study sites can be obtained	128
Eligibility criteria	10	Inclusion and exclusion criteria for participants. If applicable, eligibility criteria for study centres and individuals who will perform the interventions (eg, surgeons, psychotherapists)	144-171
Interventions	11a	Interventions for each group with sufficient detail to allow replication, including how and when they will be administered	210-278
	11b	Criteria for discontinuing or modifying allocated interventions for a given trial participant (eg, drug dose change in response to harms, participant request, or improving/worsening disease)	414-419
	11c	Strategies to improve adherence to intervention protocols, and any procedures for monitoring adherence (eg, drug tablet return, laboratory tests)	405-413

For peer review only

	11d	Relevant concomitant care and interventions that are permitted or prohibited during the trial	274-278 and 420-431
Outcomes	12	Primary, secondary, and other outcomes, including the specific measurement variable (eg, systolic blood pressure), analysis metric (eg, change from baseline, final value, time to event), method of aggregation (eg, median, proportion), and time point for each outcome. Explanation of the clinical relevance of chosen efficacy and harm outcomes is strongly recommended	292-357
Participant timeline	13	Time schedule of enrolment, interventions (including any run-ins and washouts), assessments, and visits for participants. A schematic diagram is highly recommended (see Figure)	Figure 1 and Table 1
Sample size	14	Estimated number of participants needed to achieve study objectives and how it was determined, including clinical and statistical assumptions supporting any sample size calculations	190-202
Recruitment	15	Strategies for achieving adequate participant enrolment to reach target sample size	127-143
**Methods: Assignment of interventions (for controlled trials)**			
Allocation:			
Sequence generation	16a	Method of generating the allocation sequence (eg, computer-generated random numbers), and list of any factors for stratification. To reduce predictability of a random sequence, details of any planned restriction (eg, blocking) should be provided in a separate document that is unavailable to those who enrol participants or assign interventions	203-209
Allocation concealment mechanism	16b	Mechanism of implementing the allocation sequence (eg, central telephone; sequentially numbered, opaque, sealed envelopes), describing any steps to conceal the sequence until interventions are assigned	203-209

Implementation	16c	Who will generate the allocation sequence, who will enrol participants, and who will assign participants to interventions	203-209
Blinding (masking)	17a	Who will be blinded after assignment to interventions (eg, trial participants, care providers, outcome assessors, data analysts), and how	207-209
	17b	If blinded, circumstances under which unblinding is permissible, and procedure for revealing a participant's allocated intervention during the trial	N/A
**Methods: Data collection, management, and analysis**			
Data collection methods	18a	Plans for assessment and collection of outcome, baseline, and other trial data, including any related processes to promote data quality (eg, duplicate measurements, training of assessors) and a description of study instruments (eg, questionnaires, laboratory tests) along with their reliability and validity, if known. Reference to where data collection forms can be found, if not in the protocol	360-402
	18b	Plans to promote participant retention and complete follow-up, including list of any outcome data to be collected for participants who discontinue or deviate from intervention protocols	405-413
Data management	19	Plans for data entry, coding, security, and storage, including any related processes to promote data quality (eg, double data entry; range checks for data values). Reference to where details of data management procedures can be found, if not in the protocol	468-484
Statistical methods	20a	Statistical methods for analysing primary and secondary outcomes. Reference to where other details of the statistical analysis plan can be found, if not in the protocol	432-444
	20b	Methods for any additional analyses (eg, subgroup and adjusted analyses)	432-444
	20c	Definition of analysis population relating to protocol non-adherence (eg, as randomised analysis), and any statistical methods to handle missing data (eg, multiple imputation)	432-444
**Methods: Monitoring**			

For peer review only - http://bmjopen.bmj.com/site/about/guidelines.xhtml

Data monitoring	21a	Composition of data monitoring committee (DMC); summary of its role and reporting structure; statement of whether it is independent from the sponsor and competing interests; and reference to where further details about its charter can be found, if not in the protocol. Alternatively, an explanation of why a DMC is not needed	458-463
	21b	Description of any interim analyses and stopping guidelines, including who will have access to these interim results and make the final decision to terminate the trial	458-463
Harms	22	Plans for collecting, assessing, reporting, and managing solicited and spontaneously reported adverse events and other unintended effects of trial interventions or trial conduct	464-467
Auditing	23	Frequency and procedures for auditing trial conduct, if any, and whether the process will be independent from investigators and the sponsor	458-463
**Ethics and dissemination**			
Research ethics approval	24	Plans for seeking research ethics committee/institutional review board (REC/IRB) approval	486-488
Protocol amendments	25	Plans for communicating important protocol modifications (eg, changes to eligibility criteria, outcomes, analyses) to relevant parties (eg, investigators, REC/IRBs, trial participants, trial registries, journals, regulators)	489-491
Consent or assent	26a	Who will obtain informed consent or assent from potential trial participants or authorised surrogates, and how (see Item 32)	173-175
	26b	Additional consent provisions for collection and use of participant data and biological specimens in ancillary studies, if applicable	N/A
Confidentiality	27	How personal information about potential and enrolled participants will be collected, shared, and maintained in order to protect confidentiality before, during, and after the trial	470-474
Declaration of interests	28	Financial and other competing interests for principal investigators for the overall trial and each study site	518

For peer review only - http://bmjopen.bmj.com/site/about/guidelines.xhtml

Access to data	29	Statement of who will have access to the final trial dataset, and disclosure of contractual agreements that limit such access for investigators	481-484
Ancillary and post-trial care	30	Provisions, if any, for ancillary and post-trial care, and for compensation to those who suffer harm from trial participation	Post-trial care N/A<br><br>Compensation: Sponsor
Dissemination policy	31a	Plans for investigators and sponsor to communicate trial results to participants, healthcare professionals, the public, and other relevant groups (eg, via publication, reporting in results databases, or other data sharing arrangements), including any publication restrictions	491-494
	31b	Authorship eligibility guidelines and any intended use of professional writers	N/A
	31c	Plans, if any, for granting public access to the full protocol, participant-level dataset, and statistical code	491-494
**Appendices**			
Informed consent materials	32	Model consent form and other related documentation given to participants and authorised surrogates	Available upon requested and on trial registry
Biological specimens	33	Plans for collection, laboratory evaluation, and storage of biological specimens for genetic or molecular analysis in the current trial and for future use in ancillary studies, if applicable	N/A, as per NHS procedures

For peer review only - http://bmjopen.bmj.com/site/about/guidelines.xhtml

*It is strongly recommended that this checklist be read in conjunction with the SPIRIT 2013 Explanation & Elaboration for important clarification on the items. Amendments to the protocol should be tracked and dated. The SPIRIT checklist is copyrighted by the SPIRIT Group under the Creative Commons "Attribution-NonCommercial-NoDerivs 3.0 Unported" license.

For peer review only
